# Many-Particle Li Ion Dynamics in LiMPO_4_ Olivine Phosphates (M = Mn, Fe)

**DOI:** 10.1021/acs.jpcc.2c02013

**Published:** 2022-07-22

**Authors:** Timothy Flack, Samuel A. Jobbins, Salah Eddine Boulfelfel, Stefano Leoni

**Affiliations:** † Materials Discovery Group, School of Chemistry, Cardiff University, C10 3AT Cardiff, U.K.; ‡ School of Medicine, Cardiff University, C10 3AT Cardiff, U.K.; § School of Chemical and Biomolecular Engineering, Georgia Institute of Technology, Atlanta, Georgia 30332-0100, United States

## Abstract

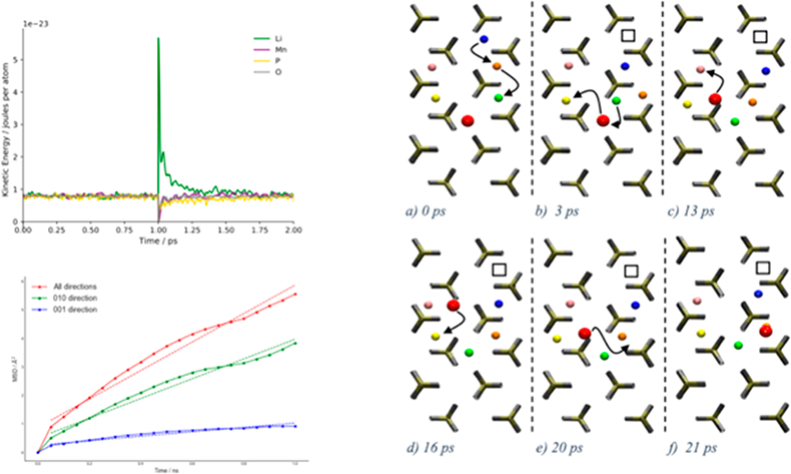

LiMPO_4_ (M = Mn, Fe) olivine phosphates are important materials for battery applications due to their stability, safety, and reliable recharge cycle. Despite continuous experimental and computational investigations, several aspects of these materials remain challenging, including conductivity dimensionality and how it maps onto Li pathways. In this work, we use a refined version of our finite temperature molecular dynamics “shooting” approach, originally designed to enhance Li hopping probability. We perform a comparative analysis of ion mobility in both materials, focused on many-particle effects. Therein, we identify main [010] diffusion channels, as well as means of interchannel couplings, in the form of Li lateral [001] hopping, which markedly impact the overall mobility efficiency as measured by self-diffusion coefficients. This clearly supports the need of many-particle approaches for reliable mechanistic investigations and for battery materials benchmarking due to the complex nature of the diffusion and transport mechanisms.

## 1 Introduction

The need for clean, efficient, and fast energy storage has grown exponentially over the past few decades and has been driven predominantly by global warming, the concurrent diminishing fossil fuel resources, and the mounting demand for portable electronics and grid storage systems.^1,2^ A significant quantity of research has been directed toward polyanion compounds with the general formula LiMXO_4_, where M = Fe, Co, Mn, or Ni and X = P, Mo, W, or S. One polyanion compound that has garnered a substantial amount of attention is triphylite or lithium ferrophosphate, LiFePO_4_ (LFP). LFP belongs to the olivine family of lithium orthophosphates, whose orthorhombic crystal structure involves corner-sharing FeO_6_ octahedra and edge-sharing LiO_6_ octahedra, which are linked together by PO_4_ tetrahedra. LFP exhibits a discharge potential of ∼3.4 V versus lithium, a theoretical capacity of 170 mAh g^–1^, and no obvious capacity fading after several hundreds of cycles.^3^ The excellent thermal stability of the O–P bond means there is no significant structural rearrangement upon intercalation/deintercalation of Li^+^ ions and no structural degradation upon overcharging. This is typified by its commercialization in 2006 and by the fact that it is now commonplace among battery devices. Much research has been directed toward improving the electrochemical performance on LFP^3−7^ by using a combination of synthesis methods, including nanoparticulate coating with electronically conductive phase such as carbon,^8^ to enhance on LFP’s intrinsic poor electronic and ionic conductivity. Fundamental knowledge of the Li^+^ ion diffusion process on the atomic scale is critical to determining the governing factors of the electrochemical behavior, including diffusion pathways and, importantly, their coupling to electronic degrees of freedom.

Experimental studies by Franger *et al*.^9^ estimated Li^+^ ion diffusion in LiFePO_4_ at 10^–14^–10^–13^ cm^2^ s^–1^. The low figure of experimental diffusion coefficients has been attributed to a series of material properties including two phase coexistence (Li_*x*_FePO_4_–Li_1–*x*_FePO_4_) during charge/discharge, a large miscibility gap between the end-members at room temperature,^10,11^ the unidimensional nature of Li^+^ ion diffusion,^12,13^ and phase transformation during lithiation.^14,15^ Puzzlingly, theoretical values predict relatively fast bulk Li^+^ ion diffusion in the range of 10^–9^–10^–8^ cm^2^ s^–1^.^13,16^ Muon spectroscopy^17^ places self-diffusion in the range (1.8–2.3) × 10^–10^ cm^2^ s^–1^, closer to computed values, pinpointing the role of the experimental techniques in determining the range of diffusion coefficients. The understanding of the Li^+^ ion diffusion mechanism and relevant pathways within such materials is therefore a top priority to the development of high-performance battery materials.

Early DFT studies^13^ indicated Li ions diffuse in one-dimensional channels along [010] and predicted very low activation barriers (∼0.1–0.2 eV) in comparison to experiment. Later studies based on interatomic potentials^18^ calculated activation barriers (∼0.5 eV) closer to the experimental values^19^ (0.54–0.63 eV) while also suggesting lithium ions follow a nonlinear trajectory along [010], further supported by DFT^13,20^ and neutron diffraction^12^ studies.

Because of the larger distance between adjacent channels (>4.5 Å) and the higher activation barrier of [001] translocations in comparison to [010], theoretical studies support one-dimensional [010] Li^+^ ion diffusion in the ordered structure of LiFePO_4_.^13,20^ However, measurements by Amin *et al*.^21^ on single crystals indicated that Li^+^ conductivity is effectively two-dimensional. Furthermore, antisite defects generated by swapping Li^+^ and Fe^2+^ ions markedly affect the diffusion mechanism and dimensionality in LiFePO_4_. First-principles calculations^22^ and bond valence methods^23^ show that the formation of Li^+^/Fe^2+^ antisite defects enables diffusion across [010] channels already at low defects concentration (1–3%), corresponding to as-synthesized LiFePO_4_.^24,25^

Static calculations typically map mechanisms onto minimum-energy paths over a double-well potential barrier separating adjacent Li sites. By use of *ab initio* molecular dynamics simulations, cross-channel migration was observed^26^ upon formation of Li^+^/Fe^2+^ antisite defects. The activation energy for cross-channel migration Li^+^ ion moving along a [010] channel with an antisite Fe^2+^ ion was calculated to be 0.491 eV, which was lower than and therefore competitive with in-channel jumps (0.70–0.74 eV). Although Li/Fe antisite defects can negatively impact ion mobility by blocking diffusion along [010] channels, they also open pathways across channels.^19^ In a scenario of competitive pathways, molecular dynamics (MD) simulations allow in principle for a detailed mechanistic analysis of Li pathways characterized by multiple and complex particle translocation events, including particle–particle correlations and long sequences of intersite Li^+^ jumps.^26^ However, simulating diffusion using straightforward MD requires overdriving temperature to overcome activation barriers, a factor that can be expected to reduce the simulation efficiency and blur mechanistic details.

To extend investigation beyond single particle translocation,^27^ we proposed the “shooter” method in conjunction with finite temperature MD, targeting many-particle Li^+^ ion diffusion dynamics under consideration of all degrees of freedom. This approach was able to yield enhanced atomistic details of Li^+^ diffusion mechanisms within LiFePO_4_. Frenkel defect formation was critical to the mechanism of particle diffusion, including [010] channel activation via cross-channel [001] jumps as an important part of the reaction coordinate of Li^+^ diffusion, while Li^+^/Fe^2+^ antisite defects introduced additional pathways for Li^+^ ion migration.

In this work we present a complete mechanistic analysis of ionic translocation within two benchmark materials, LiFePO_4_ and LiMnPO_4_ (LMP) based on finite temperature many-particle “shooter” molecular dynamics. Although advantageous in terms of redox potential, LMP bears inferior electrochemical performances compared to LiFePO_4_, exhibiting poorer Li ion diffusion and low electronic and ionic conductivities.^19,28^ This is attributed to Jahn–Teller instability of d^4^ Mn^3+^ ions, which entails MnO_6_ octahedra distortion as well as localization of the hole polaron.^29−32^ Static Li migration calculations predicted an even higher barrier for LiMnPO_4_ (0.62 eV) compared to LiFePO_4_ (0.55 eV).^27^ Also, [010] channels are indicated as only viable pathways for ionic translocation. Understanding the mechanism of diffusion, including relevant pathways and what are major hindrances to ion mobility is therefore key toward better energy storage materials.

The computational methodology presented in this work is an improved version of the simulation scheme presented in our previous work.^33^ To increase the efficiency of sampling Li^+^ hopping events during simulations, we introduced systematic changes to control the shooting regime. This allowed to reduce the temperature used for a shooting event or momenta redistribution and helped in increasing the stability of simulations by shortening the time needed to equilibrate the system after each shooting event. These important modifications were implemented and tested for LiFePO_4_ as discussed below and then applied to study Li ions diffusion mechanism in pristine LiMnPO_4_ and in the presence of antisite defects.

## 2 Methods and Models

A simulation box consisting of 1120 atoms (2 × 4 × 5 supercell) was used for both LiFePO_4_ and LiMnPO_4_. All molecular dynamics simulations were performed by using CP2K package^34^ in NPT ensemble with periodic boundary conditions. Newton’s equations of motion were integrated by using a velocity-Verlet algorithm with a time step of 0.2 fs. Nonbonded van der Waals interactions were modeled with a Buckingham potential based on a shell model for M^2+^ and O^2–^ ions, allowing for polarization effects.^27,33^ Interatomic potentials for LiFePO_4_ and LiMnPO_4_ from Fisher *et al*.^27^ were used in this work as they have been shown to accurately reproduce the experimentally observed equilibrium orthorhombic structure and defect energetics.

To enhance the frequency of Li^+^ ions hopping without exceedingly warming up the nondiffusing part of the system, we used a modified version of the simulation scheme utilized for Li^+^ mobility in LiFePO_4_.^33^ The acceleration of Li^+^ ions dynamics is achieved by applying momenta redistribution, so-called “shooting” moves to all mobile particles in the system, while keeping their positions unchanged, as discussed in section S1 of the Supporting Information. These perturbations were applied under conservation of total linear, angular momentum, and kinetic energy. The relaxation time from velocity autocorrelation function of Li^+^ ions (see Figure S3) based on independent standard molecular dynamics simulation was used to determine the optimal time interval between successive shooting events. At least 0.5 ps was required to allow Li^+^ ions equilibration after a shooting move and to ensure dynamical decorrelation. Another parameter controlling the magnitude of shooting moves was the half-width of the Gaussian smearing function applied to Li^+^ atoms momenta. Values within the interval 10^–5^–10^–2^ Å/fs, delimiting *weak* and *strong* momenta perturbations (*low* and *high* shooting regimes), were tested. In all our simulations, a time interval of 2 ps between shooting moves and a half-width equal to 10^–4^ Å/fs were used, unless otherwise specified.

For Li/M antisites defect creation, randomly selected Li^+^ ions were swapped with their nearest metal atom, M. MD simulations were run for at least 0.8 ns and shooter moves applied every 0.5 ps, applying a Gaussian half-width of 10^–4^ Å^2^/fs.

These settings represented a good compromise in allowing the whole system to equilibrate after shooting moves while maintaining a good sampling of Li^+^ hopping events during the molecular dynamics simulations. We emphasize that the general mechanistic features are not affected by a specific choice the shooting moves frequency and magnitude of momenta perturbations. A detailed description of the shooter method algorithm is presented in section S1.3 of the Supporting Information.

For each system, self-diffusion coefficients were computed from a linear fit of time evolution of mean-squared displacement (MSD) to the Einstein equation ⟨*r*^2^ ⟩ = 2*dD*_s_*t* (*r* is displacement of the mobile Li ions, *d* is dimensionality of the system, and *D*_s_ is the self-diffusion constant). MSD values were averaged over multiple time origins as described in section S1.4 of the Supporting Information. Each self-diffusion constant was averaged over data from five independent runs with different initial velocity distributions.

## 3 Results

Li^+^ ion diffusion in LiFePO_4_ and LiMnPO_4_ is an activated process biased by energy barriers larger than *k*_B_*T*, even at room conditions. By use of standard molecular dynamics simulations, the frequency of Li^+^ ions hopping is therefore too low at normal conditions even for very long running times. Consequently, the derivation of a self-diffusion constant from mean-squared displacements of Li^+^ ions is not possible as shown in Figures S4 and S5. A common approach to tackle the time scale problem in similar processes is trying to increase the frequency of Li^+^ ions hopping by using high-temperature molecular dynamics simulations. However, tests for LiFePO_4_ and LiMnPO_4_ at 700 and 1000 K did not lead to a noticeable increase in hopping frequency as evidenced by the flat root mean-squared displacements curves of Li^+^ ions shown in Figures S6–S9.

To enhance the sampling of hopping events during the diffusion process using molecular dynamics methods, we selectively coupled Li^+^ ions to a heat bath using a method comparable to the stochastic scheme of an Andersen thermostat. While the temperature of the whole system is still controlled through the thermostat of the NPT ensemble at 700 K, Li^+^ ions are systematically “warmed up” by transferring kinetic energy from the nondiffusing part of the system as described in sections 2 and S1. The trajectories generated by using this simulation methodology were used for diffusion mechanism analysis in pristine materials and in the presence of defects, for both LFP and LMP.

### 3.1 Many-Particle Diffusion Mechanism in Ordered LiFePO_4_ and LiMnPO_4_

**Figure 1:**
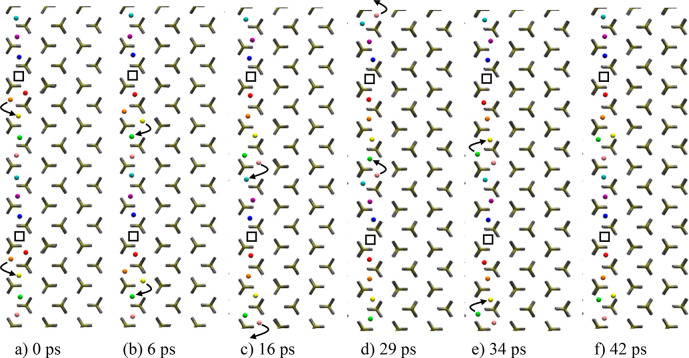
LiMnPO_4_ (low shooting regime). Snapshots of a representative diffusion mechanism within a single [010] channel (vertically). Li^+^ ions are individually colored (same color for periodic images). (a) After Frenkel defect creation, double Li occupancy begins to migrate down the channel and away from the vacancy, minimizing the chance of recombining (a). The column of Li^+^ atom remain activated by this separated Frenkel pair, with single hopping events occurring sporadically (b, c, d, e). After 42 ps, the Frenkel pair remains separated (f).

The overall mechanism entails sequences of ion translocations taking place principally along [010] for both LiFePO_4_ and LiMnPO_4_. Ionic mobility is initiated by Frenkel defects creations followed by Li cations migration as illustrated in Figures 1 and S10 for LiMnPO_4_ and LiFePO_4_, respectively. An interstitial double occupancy and a corresponding vacancy formed an initial Frenkel pair prompted either by a single jump (Figure 1a) or as a result of multiple, consecutive Li^+^ ion hopping events (Figure S10a). In Figure 1, an interstitial Li^+^ ion drifted away from the vacancy via single jumps along [010] (Figure 1a–f), while the vacancy was still at the initial position after 42 ps in LiMnPO_4_. In LiFePO_4_, Frenkel defects were created in the same [010] channel, and the corresponding vacancies propagated along with Li^+^ ions as shown in Figure S10b–d. The atomistic details of ionic mobility in LiFePO_4_ and LiMnPO_4_ revealed from trajectories illustrated in Figures 1 and S10 clearly highlighted a complex diffusion mechanism involving multiple interacting particles—one that cannot be reduced to single Li^+^ ion hopping between adjacent empty sites.

**Figure 2:**
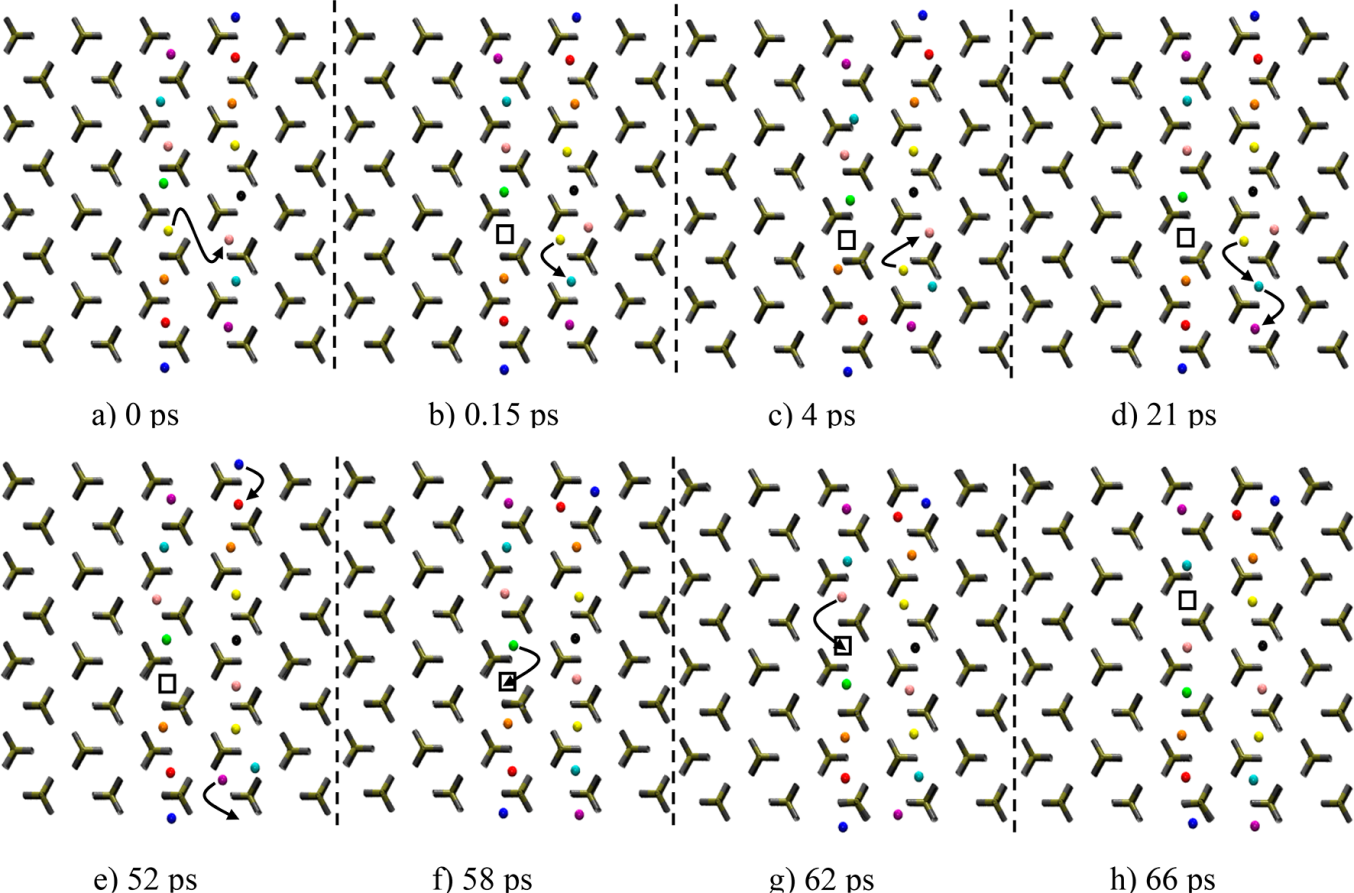
LiMnPO_4_ (low shooting regime). Snapshots of a [001] cross-jump (horizontal) and subsequent events. Coloring distinguishes Li^+^ ions within [010] channels (vertical), except for periodic images. Colors in adjacent channels are unrelated. After an initial cross-jump (a), the channels are activated in their Li mobility, enabling diffusion due to the presence of a vacancy in one channel and a double occupancy in another (b–h), which are unlikely to recombine due to infrequent recrossings (none was observed).

Another important aspect of ionic mobility in LiFePO_4_ and LiMnPO_4_ is Li^+^ ion jumps along [001]. On the basis of single particle activation energies (LiMPO_4_: M = Fe 2.89 eV, M = *M*_n_ 2.83 eV^1^), Li^+^ hopping along the *c*-axis was considered unlikely. Using our shooter method, we identified sequences involving cross-channel jumps along [001] as shown in Figures 2 and S12 for LiMnPO_4_ and LiFePO_4_, respectively. A single Li^+^ ion hop between adjacent channels resulted in the formation of a Frenkel defect. This created an excess charge around the double-occupancy site (yellow and pink Li^+^ ions in Figure 2b) and a corresponding charge deficiency was introduced around the vacancy left beyond (empty square in Figure 2b). Once created, the double occupancy is rapidly relocated within the [010] channel (Figure 2b,c, from pink/yellow to yellow/light blue Li pairs), without recrossing events. Particle and vacancy migration mostly occurred as the result of elementary single-particle migration steps (see Figure 2 (LMP) and Figure S12 (LFP)).

**Figure 3:**
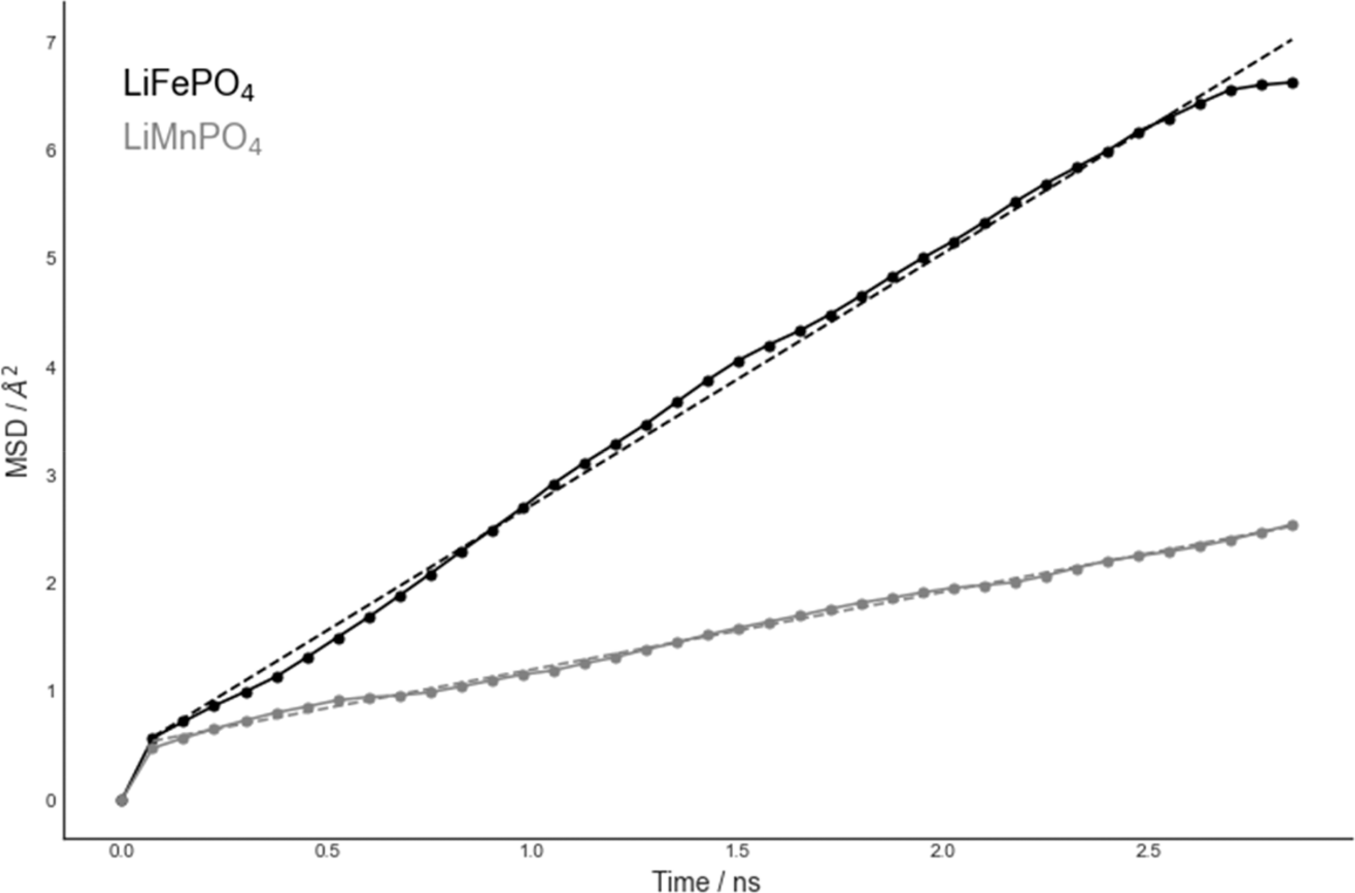
Li^+^ ions MSD vs time in LiFePO_4_ (black line, *D*_s_ = (3.87 ± 0.19) × 10^–8^ cm^2^ s^–1^) with ω_f_ = 0 ns, ω_t_ = 3.7 ns, and ω_s_ = 75 ps, and in LiMnPO_4_ (gray line, *D*_s_ = *D*_s_ = (1.19 ± 0.05) × 10^–8^ cm^2^ s^–1^) with ω_f_ = 0 ns, ω_t_ = 3.25 ns, and ω_s_ = 75 ps. Both curves were averaged over multiple time origins.

MSD curves were obtained for defect-free LiFePO_4_ and LiMnPO_4_ systems as shown in Figure 3. Our simulation scheme allowed to reduce the amplitude of momenta modifications without altering either the diffusion mechanism or the derived self-diffusion coefficients as demonstrated by comparison between low and high shooting regimes in Figures S13 and S15 for LiFePO_4_ (*D*_s_^low^/*D*_s_^high^ = 1.06). The computed Li-ion self-diffusion coefficient in LiFePO_4_ was (3.87 ± 0.19) × 10^–8^ cm^2^ s^–1^, 1 order of magnitude slower in comparison to our previous results^2^ and in better agreement with predictions in the range of 10^–9^–10^–8^ cm^2^ s^–1^ from other theoretical studies.^13,16^ In comparison to experimental studies values in the range of 10^–17^–10^–11^ cm^2^ s^–1^,^35−37^ our results are overestimating self-diffusivity of Li^+^ ions, partially due to the bias introduced by the shooter method to speed up ionic mobility. The computed Li-ion self-diffusion coefficient in LiMnPO_4_ was (1.19 ± 0.05) × 10^–8^ cm^2^ s^–1^, ∼3 times slower in comparison to LiFePO_4_ and few orders of magnitude faster than theoretical predictions of Shi *et al.* (∼10^–14^ cm^2^ s^–1^).^38^ Experimental measurements of Kwon *et al.*(39) on LiMnPO_4_ nanoparticles reported larger diffusion coefficients in the order of 10^–12^–10^–10^ cm^2^ s^–1^.

**Figure 4:**
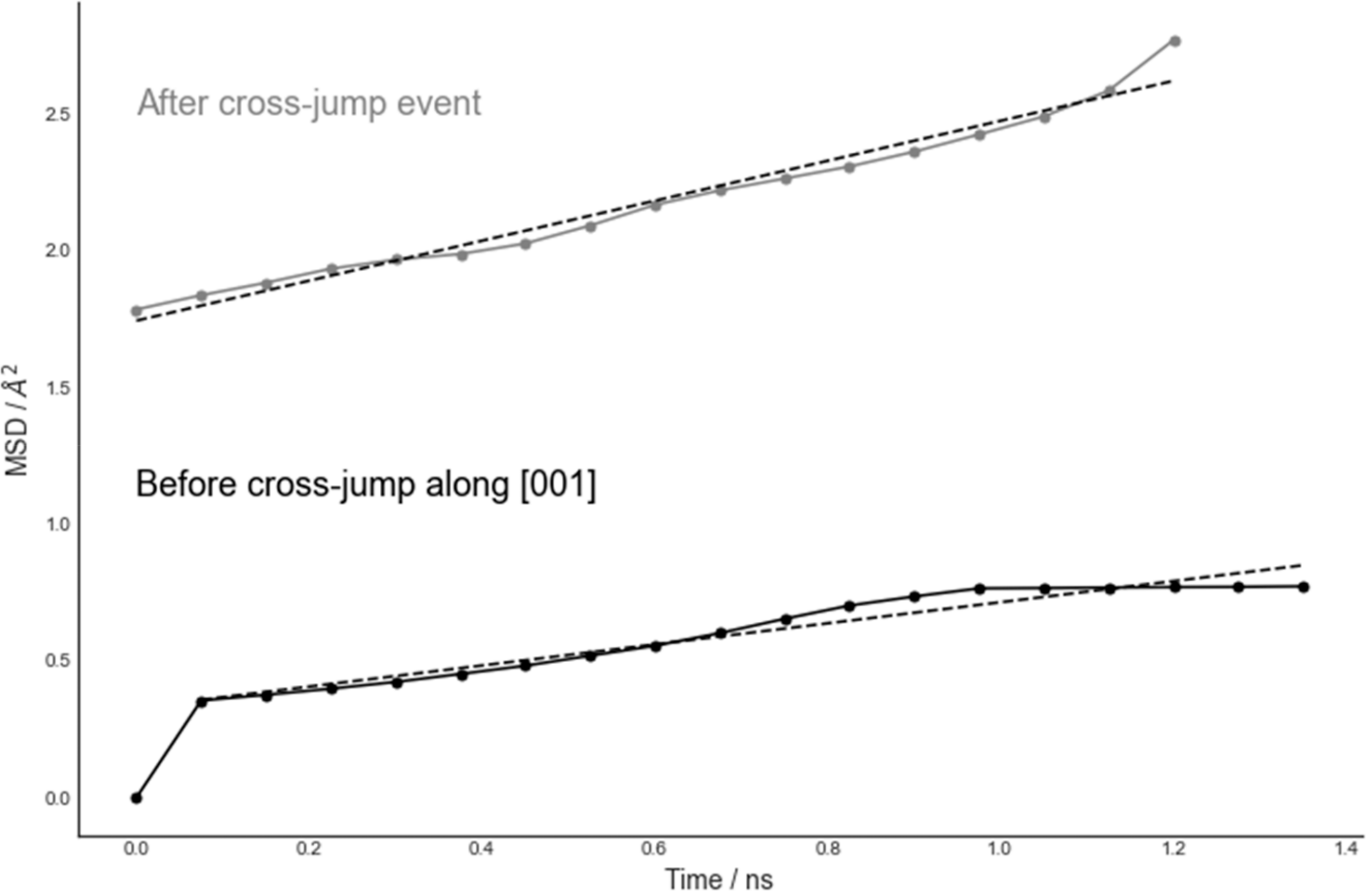
Li^+^ ions MSD vs time in LiMnPO_4_ before (black line, *D*_s_ = (6.42 ± 0.81) × 10^–9^ cm^2^ s^–1^) and after (gray line, *D*_s_ = (1.22 ± 0.42) × 10^–8^ cm^2^ s^–1^) the creation of double occupancy and vacancy in adjacent [010] channels.

The cross-channel hopping along [001], although less frequent, helped increase ionic mobility by promoting the creation of Frenkel defects. Figure 4 illustrates an increase in Li^+^ ions MSD for LiMnPO_4_ by a factor ∼5 following cross-channel jumps, suggesting an important role of many-particle charge dynamics in a 2-dimensional conductivity mechanism, despite ion mobility being dominated by diffusion along the [010] direction. The same effect on diffusion constants caused by cross-channel jumps along [100] was observed in LiFePO_4_, as shown in Figure S14 and LiMnPO_4_ in Figure S16. The two figures represent the time evolution of MSD of Li^+^ ions before and after a cross-channel jump from the same trajectory.

### 3.2 Effect of Li/M Antisite Defects (M = Fe/Mn) on Li Diffusion Mechanism in LiMPO_4_

To investigate the effect of Li^+^/M^2+^ (M = Fe/Mn) antisites on the migration along the [001] direction and the underlying mechanism, simulations were performed on both LiFePO_4_ and LiMnPO_4_. In accordance with experimental values,^24,25^ four Li^+^/M^2+^ defects were introduced into the simulation system, corresponding to a 2.5% of Li^+^ ions on antisites (4 out of 160 Li atoms in total).

**Figure 5:**
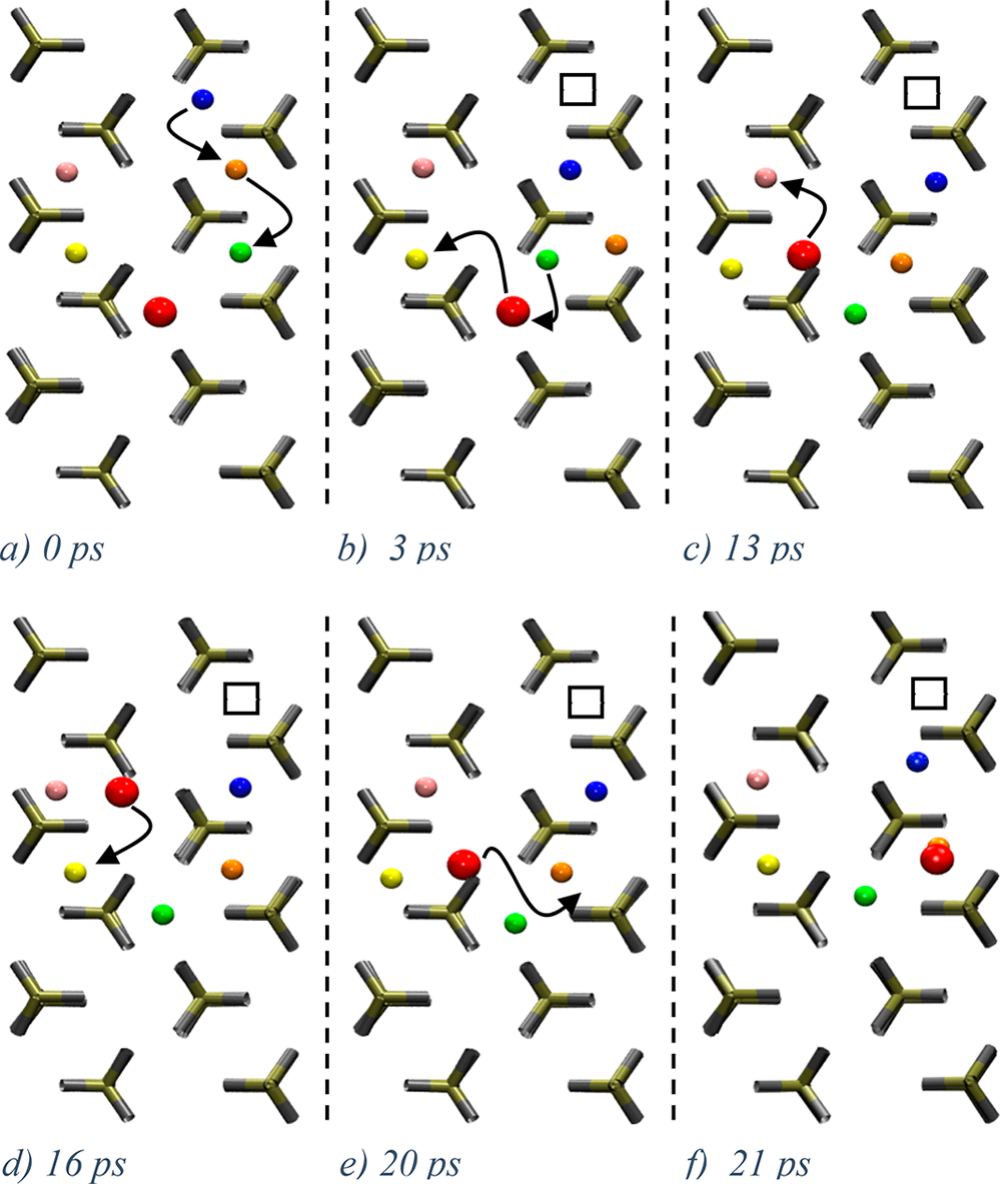
LiMnPO_4_. Snapshots illustrating two mechanisms of [001] cross-jump migration. Li^+^ are individually colored; initial antisite Li^+^ is represented as a large red atom. The sequence is initiated by (a) the combination of two single Li^+^ jumps. The [001] migration is realized through the swapping of a channel Li^+^ ion (green) and the antisite Li^+^ (red) (b). The double-occupied site migrates as the result of single particle jumps (c, d). A second mechanism of [001] migration involves a Li^+^ ion revolving past the antisite Li^+^ ion (green) (e). This second [001] migration terminates into a different channel compared to the original migration (f).

Li ion migration from channel to channel via interstitial Li^+^ ion occurred in two possible ways as shown in Figure 5. The first involved the replacement of the antisite Li^+^ ion by a pristine channel Li^+^ ion, while the second entailed a direct bypassing of the antisite Li^+^ ion by a channel Li^+^ ion. The sequence of hopping events was initiated as the result of the combination of two correlated Li^+^ ion jumps (blue and orange Li^+^ ions in Figure 5a). Local disorder within the channel was created by an interstitial double occupancy and a vacancy as shown in Figure 5b (white square). This was followed by simultaneous migration of a Li^+^ ion (green) into antisite positions and an antisite Li^+^ ion (red) into the main channel (Figure 5b,c). The vacancy and double occupancy pair are now separated in adjacent channels (Figure 5c). Rearrangement of the double-occupied site caused further ionic migration within the left channel (Figure 5c,d). Antisite recrossing was realized via a direct bypassing of the antisite Li^+^ ion (green) by the migrating Li^+^ ion (red, Figure 5e), before entering the adjacent channel (Figure 5f).

**Figure 6:**
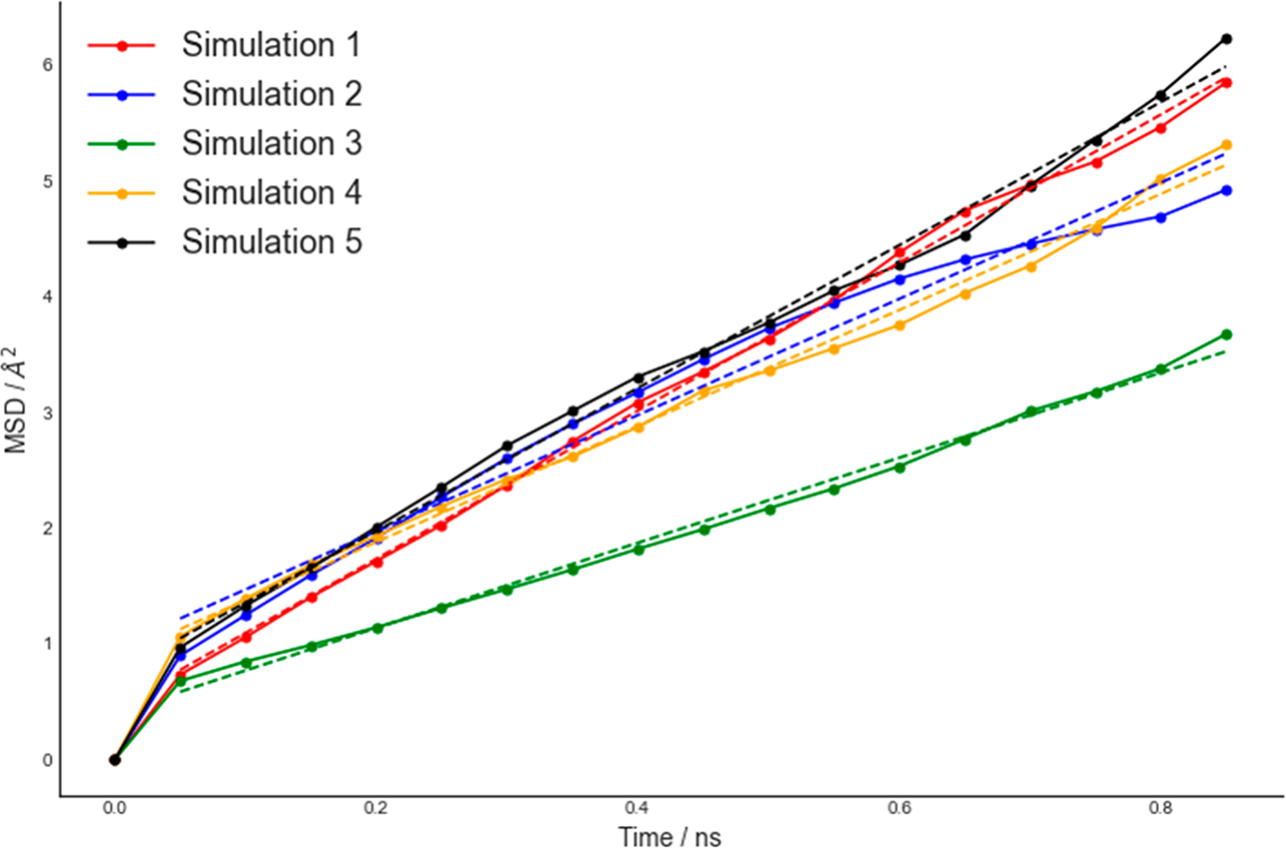
MSD vs time for all Li^+^ ions in all five simulations. MSDs are averaged over multiple time origins. ω_f_ = 0, ω_t_ = 1 ns, and ω_s_ = 50 ps.

**Figure 7:**
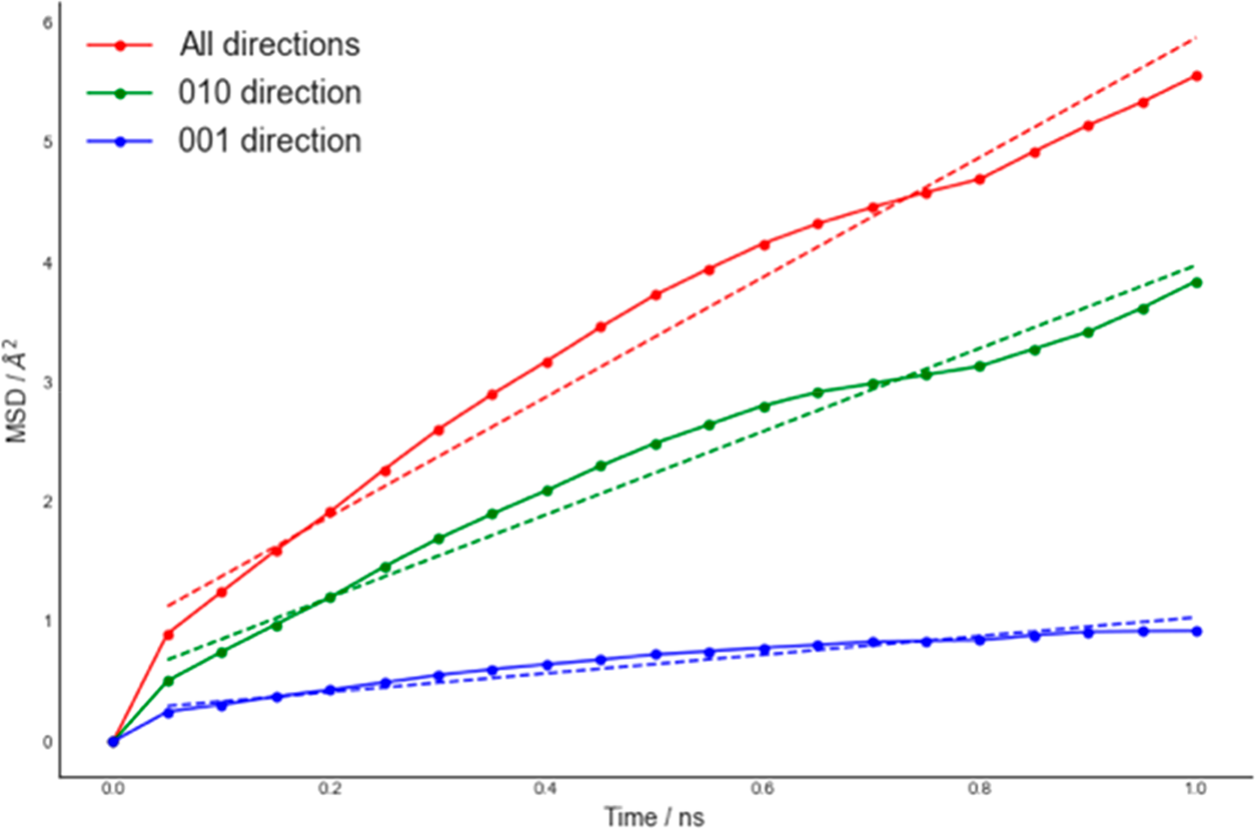
MSD vs time of Li^+^ ions for LiMnPO_4_ with antisite defects. Total MSD is shown in red, along [010] in green, and [001] in blue. Self-diffusion coefficient values are (8.76 ± 0.16) × 10^–8^ cm^2^ s^–1^ (total), (5.78 ± 0.14) × 10^–8^ cm^2^ s^–1^ ([010]), and (1.30 ± 0.12) × 10^–8^ cm^2^ s^–1^ ([001]).

This sequence of events illustrates significant correlation between mobile ions in adjacent channels and supports a two-dimensional diffusion scenario promoted by the inclusion of Li^+^/Mn^2+^ antisites. The overall effect of the antisite defects was assessed by quantifying variations in Li^+^ ions diffusivity in LiFePO_4_ and LiMnPO_4_. By use of MSD data in Figures 6 and S19, the derived Li^+^ ions self-diffusion coefficients were (8.76 ± 0.16) × 10^–8^ and (2.01 ± 0.08) × 10^–7^ cm^2^ s^–1^ in LiMnPO_4_ and LiFePO_4_, respectively. Compared to (1.19 ± 0.05) × 10^–8^ and (3.87 ± 0.19) × 10^–8^ cm^2^ s^–1^ for pristine LiMnPO_4_ and LiFePO_4_, respectively, it corresponds to an ∼7 and ∼5 times increase in Li^+^ ions diffusivity. As for LiFePO_4_, coupling between channels is enhanced upon the inclusion of Li^+^/Mn^2+^ antisites. A channel-resolved comparison of MSD in LiMnPO_4_ with antisite defects proves the activation of the [001] direction (Figure 7).

## 4 Discussion

Using the shooter enhanced sampling technique in combination with shooter molecular dynamics simulations, for both LiFePO_4_ and LiMnPO_4_ the main diffusion pathways are in the [010] direction (easy axis). Migrations along the [001] axis for both materials were also observed, albeit as minority component in the overall diffusive process, indicating a coupling between adjacent [010] channels, which markedly enhances the measured diffusion constant. This coupling is a result of many-particle dynamics and remains elusive to static calculations based on defect energies.

General mechanistic features of [010] diffusion for both LiFePO_4_ and LiMnPO_4_ are the following:

- Diffusion is initiated by the formation of a Frenkel defect within easy [010] channels.
- Frenkel defects introduce disorder within channels in the form of Li^+^ double-occupied cages and a corresponding vacancy localized at some distance, which was found to be larger for LiFePO_4_.
- Frenkel pairs are mobile species that can migrate due to single particle events (hopping into adjacent channel sites) or a combination of events (extended hopping).

Single-particle potential energy calculations^27^ have shown diffusion between channels/across channel boundaries to have a significantly higher activation energy in comparison to diffusion within “easy” channels. The shooter method has shown that in a many-particle system collective dynamics is more than the sum of isolated intersite hopping events. Although migration along [001] represents a small fraction of the reaction coordinate, it has significant implications. There is indeed experimental evidence that also suggests the [001] direction to be an active direction of diffusion within LiFePO_4_.^21^ No corresponding experiments suggest that [001] migration is part of the reaction coordinate of Li^+^ diffusion within LiMnPO_4_, which we demonstrate here computationally. Accordingly, LiMPO_4_ diffusion should be better characterized as 2D rather than restricted to easy channels only.

Migration along [001] also implies Frenkel pair mobility within [010] channels, but with a subtle difference. The result of a cross-channel jump (along [001]) is the formation of Frenkel pairs *across* channels, effectively introducing an additional diffusive dimension. One channel possesses an excess charge, manifested a double-occupied site, while an adjacent one hosts a corresponding vacancy. It has already been established that Frenkel pairs are more mobile and rapidly migrate within channels. Once they begin to migrate within separate channels, the Frenkel pair has a vanishing recombination probability. In fact, no recombination was observed during our MD simulations. This way, diffusion is permanently affected.

Self-diffusion coefficients were estimated for both materials by calculating multiple-reference Li^+^ ions MSD via the Einstein equation. Calculated values are consistent with other theoretical studies^13,16,33^ for LiFePO_4_. Values calculated for LiMnPO_4_ were lower than for LiFePO_4_, which trendwise agrees with the literature.^38,39^

The overestimation of self-diffusion coefficients calculated from the shooter method may be in part explained by the necessary bias introduced into the system, despite low shooting regimes. However, the mechanism of Li^+^ ion diffusion has been shown to be robust even for larger perturbations. The shooter method appears therefore suitable for mechanistic investigations as it correctly predicts diffusion trends.

Previous theoretical studies^2,19,23^ have shown Li^+^/M^2+^ (M = Fe^2+^/Mn^2+^) antisite defects to provide alternative routes for diffusion between channels due to additional diffusion pathways between channels in both LiFePO_4_ and LiMnPO_4_. The shooter method indicates two distinct mechanisms for cross-channel [001] diffusion: (1) Diffusion across the channels may occur via the replacement of the antisite Li^+^ ion with a pristine channel Li^+^ ion. (2) Diffusion across the channels also occurs via direct crossover of a channel Li^+^ ion *past* and antisite Li^+^ ion, without displacement of the latter. The calculated self-diffusion coefficients reveal an overall increase in diffusion for both the [010] and [001] direction. The increase in the [001] direction can be attributed to additional pathways for diffusion introduced by the antisites. The increase in the [010] direction is interesting as antisite Fe^2+^ ions could be expected to inhibit diffusion within channels. An overall increase in the [010] direction is a consequence of increased interchannel couplings that reduces Frenkel pair recombination probability.

Shooting trajectories can be used as basis for free-energy sampling approaches, like umbrella sampling. Therein, constrained MD is used on trajectory slices, corresponding to intermediate points along the reaction coordinate. In Figures S20–S22 snapshots from constrained NVT MD are presented (168 atoms box), where force calculations are based on the GFN-xTB transferrable potential^40^ (see the Supporting Information for details). Sequences of Li^+^ ion displacements correspond to force-field simulations, including the role of Frenkel pair recombination and the role of many-particle dynamics. While the computation of an accurate free energy profile is beyond the scope of this work, the transferability of the mechanistic picture across potentials further validates our findings.

## 5 Conclusions

In this work, the shooter MD approach to collective ion dynamics was further refined and extended to the investigation of Li diffusion mechanisms in LiMnPO_4_ and LiFePO_4_. The parameter space of the shooter method was investigated by allowing for a *high* and a *low* shooting regime, corresponding to variable broadening of kinetic energy redistribution. As well as providing a more reliable calculation of diffusion constants, the mechanistic details of Li^+^ ion diffusion revealed by the shooter method remain the same regardless of the severity of the shooter perturbation. The mechanistic scenarios presented in this work support a many-particle perspective on Li ion mobility, which we argue should consistently replace any single-particle approach if a mechanistic analysis is intended. In combination with better methods of defect energy calculation,^41^ many-particle assisted hopping can provide a holistic account of ion mobility in relevant materials. The coupling role of transversal jumps across channel boundaries has a significant impact on diffusion constant moduli, on the understanding of dimensionality of diffusion within materials, and on the design of novel battery materials. While in this work we have used polarizable force fields, which do not allow for electronic degrees of freedom, the shooter method can be expected to be applicable in combination with suitable potentials, based on tight-binding or ML potentials. Additionally, force-field fitting on equilibrium properties only does not guarantee control over hopping potential energy barriers. We expect the problem of the general overestimation of diffusion constants, common to most computational approaches, to be addressable in a scenario that explicitly allows for coupling of ion dynamics with electrodynamics, which is among the current challenges of battery simulations.

## Supporting Information Available

Shooter method (kinetic energy redistribution and system response, velocity autocorrelation function of Li^+^ in LiFePO_4_ and LiMnPO_4_, methodology details), RMSD plots of finite temperature MD simulations, Li diffusion mechanisms (LiFePO_4_: low shooting regime; LiFePO_4_: high shooting regime; LiFePO_4_: low shooting regime [001] crossings), mean-squared displacements MSD (LiFePO_4_: low shooting regime; LiFePO_4_: antisite defects in the optimized shooting regime); tight-binding molecular dynamics (LiFePO_4_, LiMnPO_4_) (PDF)
