# Supplementary material for: Many-Particle Li Ion Dynamics in LiMPO4 Olivine Phosphates (M = Mn, Fe)
Source: J Phys Chem C Nanomater Interfaces. 2022 Jul 22;126(30):12339–47. doi: 10.1021/acs.jpcc.2c02013 (PMC9358648; doi:10.1021/acs.jpcc.2c02013)
Supplement: Supplementary file 1 — jp2c02013_si_001.pdf [file jp2c02013_si_001.pdf]

Many-Particle Li-Ion Dynamics in $LiMPO_4$ Olivine Phosphates (M=Fe,Mn): Supporting Information

Timothy Flack[1], Samuel A. Jobbins[2], Salah Eddine Boulfelfel[3], Stefano Leoni[1,*]

[1] Materials Discovery Group, School of Chemistry, Cardiff University, C10 3AT Cardiff, UK

[2] School of Medicine, Cardiff University, C24 Cardiff, UK

[3] Georgia Institute of Technology, School of Chemical and Biomolecular Engineering, Atlanta, GA 30332-0100, USA

[*] Electronic Address: leonis@cf.ac.uk

Table of Contents

S1. Shooter Method ***3***

S1.1 Kinetic Energy Redistribution and System Response **3**

S1.2 Velocity Autocorrelation Function of Li^+ in $LiFePO_4$ and $LiMnPO_4$ **5**

S1.3 Methodology **6**

S1.4 Calculation of Mean-Squared Displacements **8**

S2. RMSD Plots of Finite Temperature MD simulations ***8***

S3. Li Diffusion Mechanisms ***11***

S3.1 $LiFePO_4$ – Low Shooting Regime **11**

S3.2 $LiFePO_4$ – High Shooting Regime **12**

S3.3 $LiFePO_4$ – Low Shooting Regime ([001] crossings) **13**

S4. Mean Squared Displacements ***14***

S4.1 $LiFePO_4$ – Low Shooting Regime **14**

S4.2 $LiFePO_4$ – High Shooting Regime **15**

S4.3 $LiMPO_4$ – "Optimized" Shooting Regime **16**

S4.4 $LiFePO_4$ – Antisite Defects (Optimized Shooting Regime) **17**

S5 Tight-Binding Molecular Dynamics ***18***

S5.1 $LiFePO_4$ **18**

S5.2 $LiMnPO_4$ **20**

References ***21***

S1. Shooter Method

S1.1 Kinetic Energy Redistribution and System Response

The shooter move selectively warms up the Li^+ ions by transferring a variable amount of kinetic energy from the host framework (MPO_4, M = Fe/Mn) to the mobile ions (Li^+). This way a clear separation of velocities, and hence kinetic energy, can be achieved. This separation is explicitly calculated in Figs.

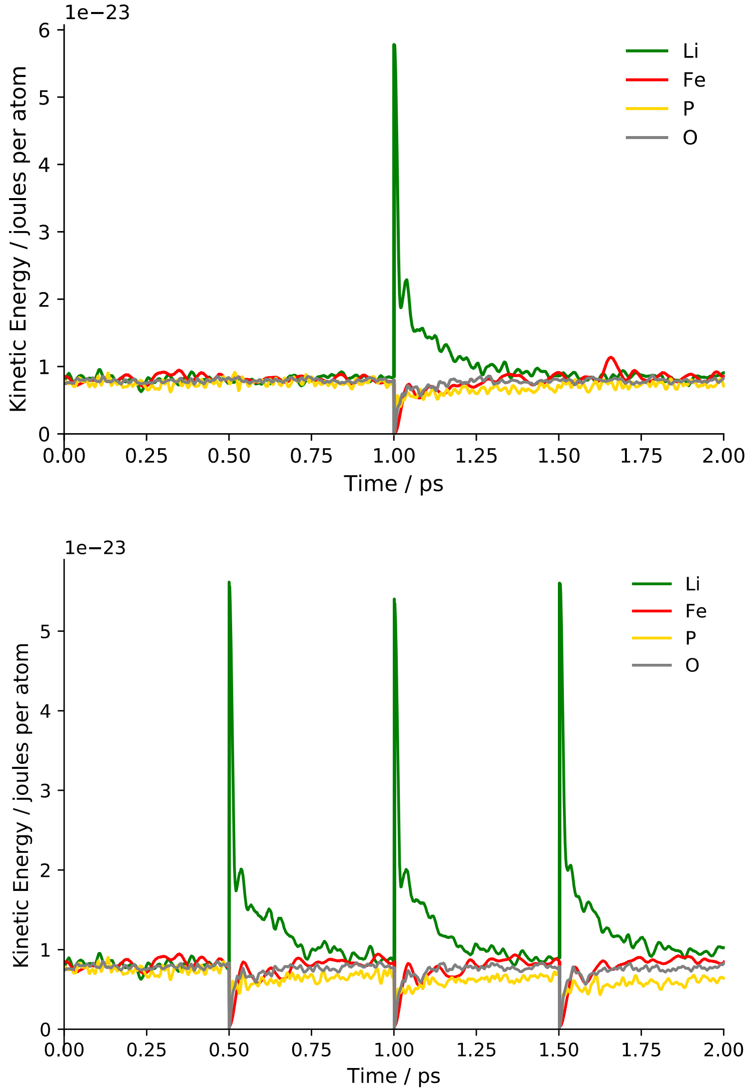

Figure S1: The effect of one 'Shooting' event on the total kinetic energies of each constituent atom type in $LiFePO_4$ (top) and the effect of multiple 'Shooting' events (bottom).

The application of the 'Shooter' algorithm generates a separation in kinetic energy between Li^+ and $FePO_4$ (Fig. S7, upper part). This separation is attained *via* the generation of a velocity

distribution not typical of the ensemble. This separation gradually decays and after approximately 0.5 ps the equilibrium distribution is recovered, up to some echoes between 1.0 and 2.0 ps from the pulse. Multiple applications of the ‘Shooter’ algorithm, every 0.5 ps as shown in Fig. S7 (lower part), establishes and partially maintains a separation, keeping therefore some amount of bias on the distribution. The same effect is achieved in applying the ‘Shooter’ algorithm to $LiMnPO_4$, as shown in Fig. S8.

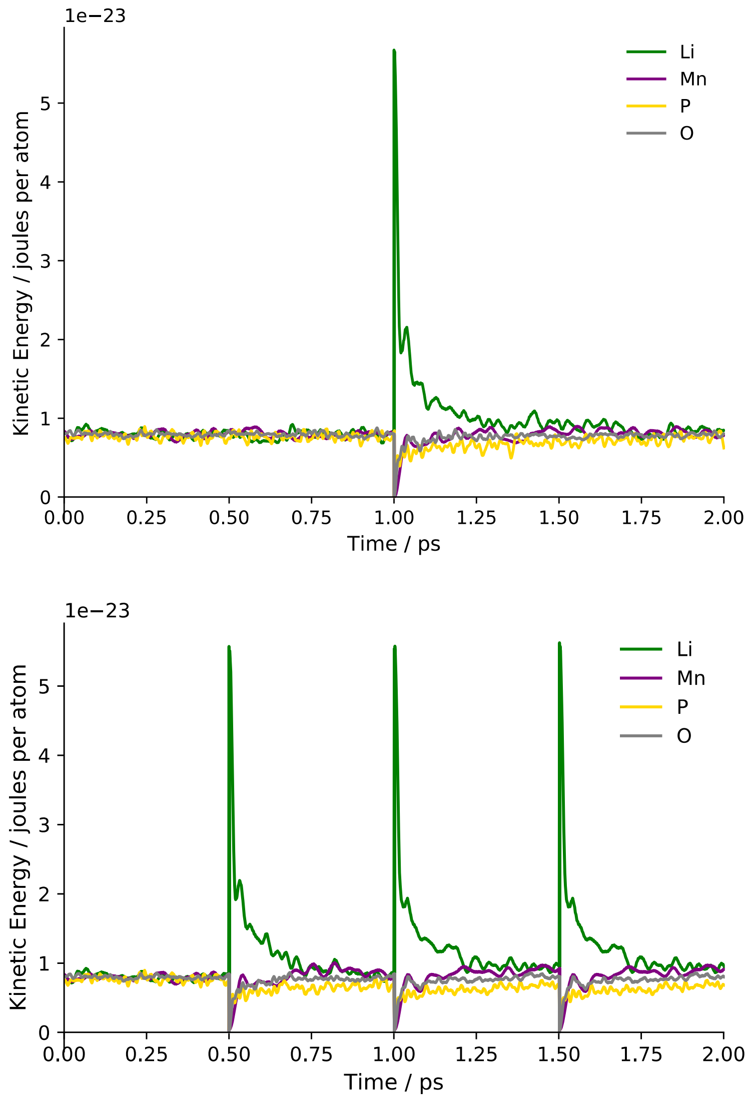

Figure S2: The effect of one ‘Shooting’ event on the total kinetic energies and their distribution on each constituent atom type in $LiMnPO_4$ (top) and the effect of multiple ‘Shooting’ events (bottom). Atoms are color-coded.

S1.2 Velocity Autocorrelation Function of Li^+ in $LiFePO_4$ and $LiMnPO_4$

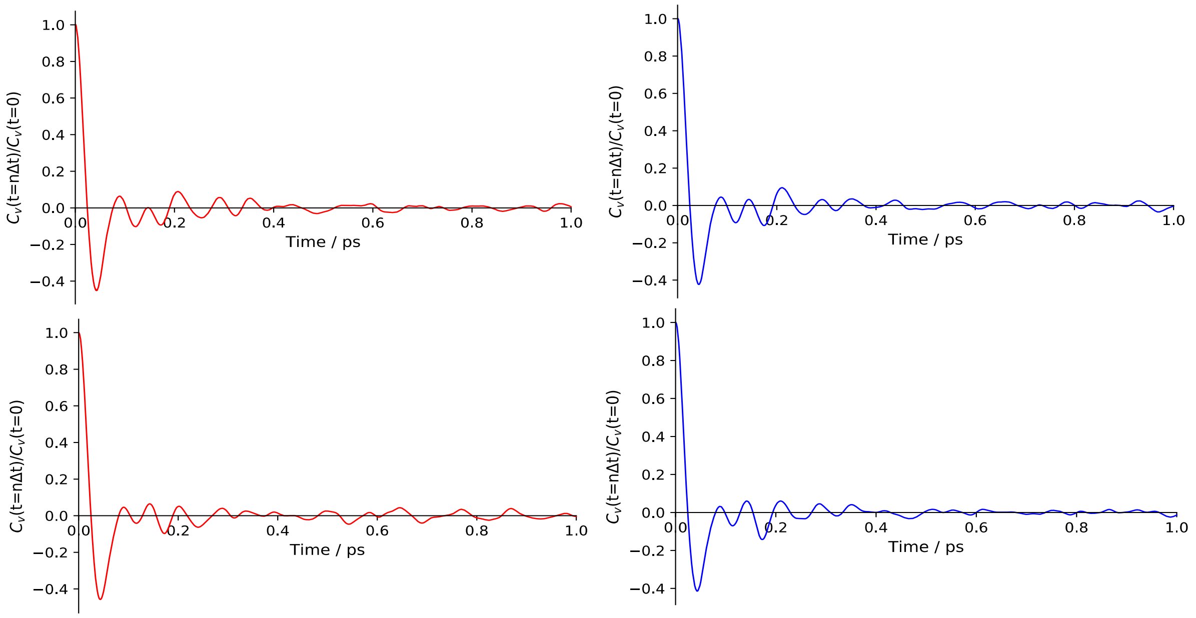

Figure S3: Normalized velocity autocorrelation function of Li^+ ions within $LiFePO_4$, (top) and $LiMnPO_4$ (bottom) for both, a MD simulation of an equilibrated structure at 700 K (left, red) and a resulting velocity distribution after a 'shooting' event (right, blue). The total simulation time was 10 ps, 10 VACF functions of 1 ps each were calculated and averaged.

From the velocity autocorrelation function of Li^+ ions in Figure S3, a time interval of 0.5 picoseconds between two successive shooting moves is enough to allow system equilibration and decorrelation of Li^+ dynamics. To understand how the shooter approach acts on the system equilibrium distributions, the VACF of equilibrium velocity distributions are shown in Fig. S3 and compared to post-'shooting' event velocity distributions for $LiMPO_4$. In both cases an initial rapid decay is observed, followed by damped oscillations around zero. After about 0.5 ps, the velocities are largely decorrelated, in both straightforward and biased simulation regimes. The VACF autocorrelation functions indicate that the shooter-perturbed system returns to equilibrium in a way that is indistinguishable from its response to spontaneous fluctuations, in line with Onsager's regression hypothesis. Our approach therefore excites specific fluctuations, while (in principle) preserving the "measurability" of a system *close enough* to equilibrium.

S1.3 Methodology

As its name suggests, this approach is a modification of the shooter algorithm used in transition path sampling, which is employed here to systematically perturbate points in phase space[33]. The following general steps describe a typical 'Shooter' move simulation used throughout this body of work:

i. A thermally equilibrated configuration was used to initialize the simulation at 700 K.

ii. A small perturbation was chosen to Li ions only, by setting a Gaussian half-width, centered on the velocity of each Li ions. An initial half-width was chosen in two different simulations regimes, as described below.

iii. The instantaneous frame temperature T_i was calculated from the total kinetic energy, $T(t) = \sum_{i=1}^{N} \frac{m_i v_i^2(t)}{k_B N_f}$. Here, the sum is calculated over all atoms of mass m_i and velocity v_i. k_B is the Boltzmann constant and N_f is the number of degrees of freedom.

iv. Velocities of Li ions are perturbed by choosing a random value from the Gaussian distribution centered around its current velocity. The new velocity distribution is accepted with probability $\min[1, \rho(x_t^{new})/\rho(x_t^{new})]$;

v. A post-perturbation temperature T_{pp} was calculated from the rescale kinetic energy.

vi. The initial temperature was restored through rescaling the velocities of all other particles by $s = \sqrt{\frac{T_i}{T_{pp}}}$.

vii. The frame is propagated for a set amount of time (at least 0.5 ps as indicated by the analysis of the vac(t), see main text for details).

viii. The Gaussian half-width is increased by a smearing factor, typically 1.0001.

ix. This process is repeated until the total simulation time reaches a target value of choice. Typical simulation times were in the order of 300-500 ps for mechanistic assessments and 3-5 ns for MSD/Diffusion Constants evaluations, as described below.

The implementation of the shooter-enhanced MD simulation was achieved as chain of shooting steps, regularly spaced in time. The VACF relaxation time served as a lower relaxation limit for the time delay between shooting events, at least 0.5 ps. The Gaussian half-width, which controls the extent of kinetic energy transfer between host framework and Li ions, was chosen within the interval [5.10^{-5}–10^{-2} Å/fs], the limits corresponding to a *low* and *high* shooting regime, respectively. Most simulations (unless otherwise indicated) were performed using a longer delay of 2 ps between shooting events. This choice was motivated by kinetic energy distribution analysis after the application of both single and sequential shooting pulses. A single pulse (Figs. S1 and S2) causes echoes in the kinetic energy fluctuations of heavier particle after 0.5 ps from shooter onset. If too closely sequenced (0.5 ps), shooting pulses would enforce specific kinetic energy separations, by partially preventing system relaxation. The choice of 2 ps represents a good compromise in keeping as close as possible to equilibrium distributions, but not too close to quench diffusive behavior. Clearly, any attempt to "optimize" shooting moves must be understood as an effort to keep the shooting perturbations as small as possible (and therefore as infrequent as possible), while maintaining steady particle motion in a linear regime, where Kubo-Green measurements can be performed. Accordingly, the aim of the shooting approach is to amplify particle translocation probability and many-particle propagation within materials as a basis for a detailed mechanistic analysis. Achieving a constant rate of hopping is instrumental to calculating diffusion coefficients based on the Einstein relation, which relates the diffusion constant to the slope of mean square displacement (MSD) as a function of time, in the long-time limit. Calculations of diffusion constants for different choices of the shooting parameters allowed in turn to assess numerical stability of the overall shooter MD approach to Li^+ ion dynamics.

S1.4 Calculation of Mean-Squared Displacements

MSD curves were averaged by selecting different reference configurations spaced by a fixed amount of time. For an interval $[\omega_f \dots \omega_t]$ and a fixed stride ω_s, an averaged MSD is computed according to:

$$MSD(\tau) = \frac{\omega_s}{\omega_t - \omega_f - \tau} \sum_{t_0 = \omega_f \; every \; \omega_s}^{\omega_t - \tau - 1} \frac{1}{N} \sum_{i=1}^{N} \left(r_i(t_0 + \tau) - r_i(t_0) \right)^2 \quad (1)$$

S2. RMSD Plots of Finite Temperature MD simulations

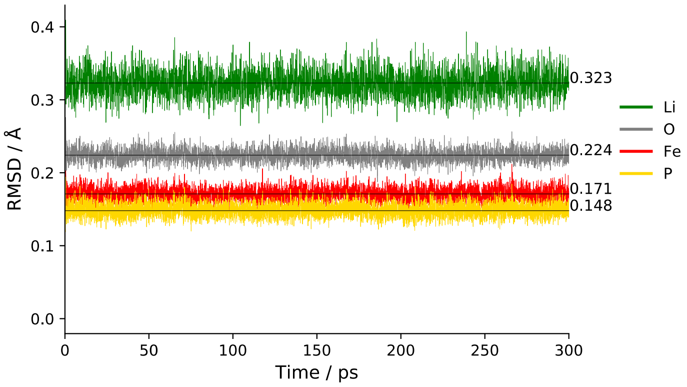

Figure S4: RMSD vs time for $LiFePO_4$ calculated from NPT MD simulation at 300 K and 1 bar (total time 300 ps). Individual atomic species are color-coded. The average RMSD value (right hand side) is represented by a black line superimposed onto each trajectory.

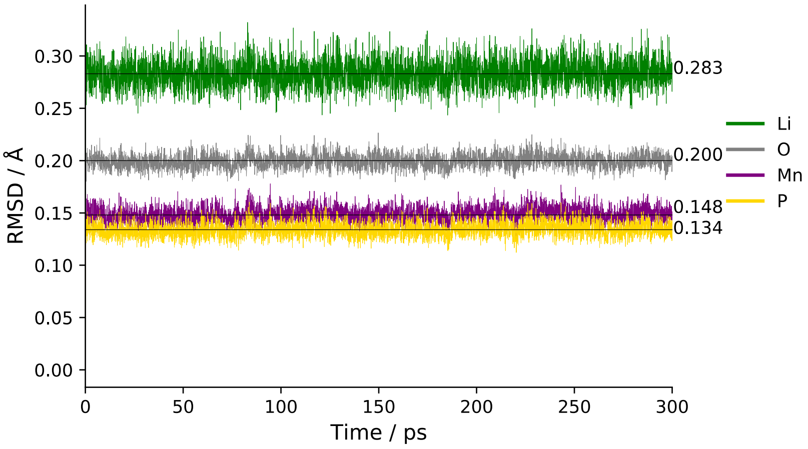

Figure S5: RMSD vs time for $LiMnPO_4$ calculated from NPT MD simulation at 300 K and 1 bar (total time 300 ps). Individual atomic species are color-coded.

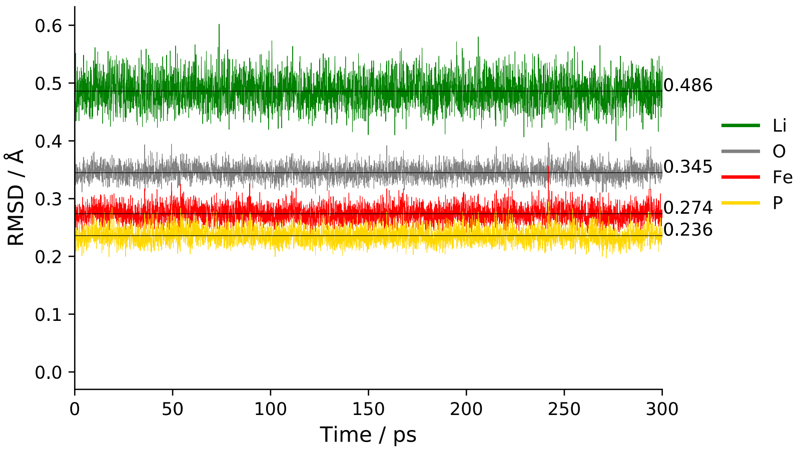

Figure S6: RMSD vs time for $LiFePO_4$ calculated from NPT MD simulation at 700 K and 1 bar (total time 300 ps). Individual atomic species are color-coded.

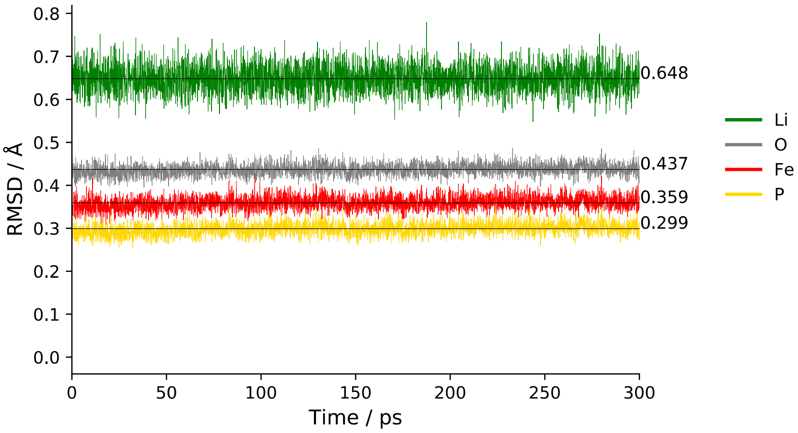

Figure S7: RMSD vs time for $LiFePO_4$ calculated from NPT MD simulation at 1000 K and 1 bar (total time 300 ps). Individual atomic species are color-coded.

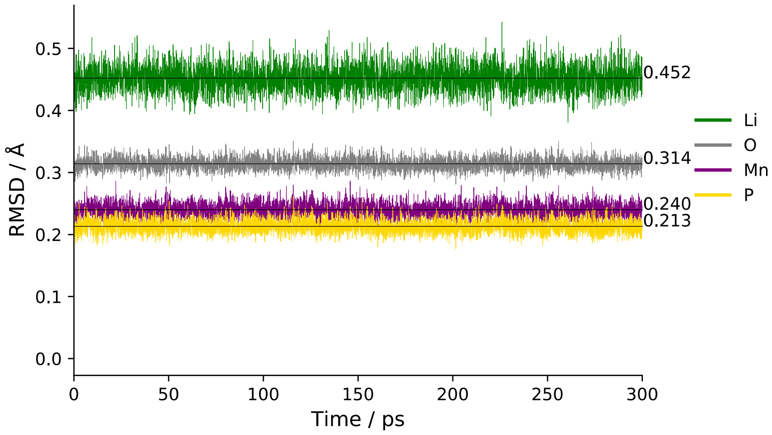

Figure S8: RMSD vs time for $LiMnPO_4$ calculated from NPT MD simulation at 700 K and 1 bar (total time 300 ps). Individual atomic species are color-coded.

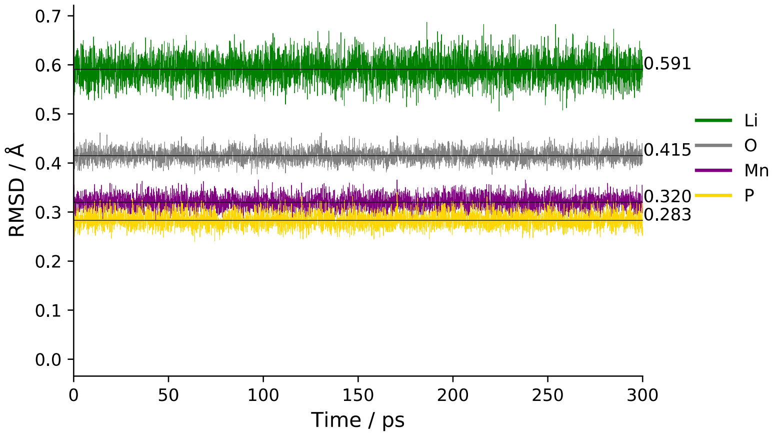

Figure S9: RMSD vs time for $LiMnPO_4$ calculated from NPT MD simulation at 1000 K and 1 bar (total time 300 ps). Individual atomic species are color-coded.

At 300 K all atoms are rattling around their equilibrium position showing no signs of diffusive behavior. Li^+ ions are more mobile in comparison to the rest of structure, displaying the highest average RMSD value (Figs. S1 to S3, values are indicated on the right-hand side). The average RMSD value for Li^+ ions increased from 0.323 Å (300 K) to 0.648 Å (1000 K), keeping a flat profile. At 300 K $LiMnPO_4$ shows no signs of diffusive behavior. All RMSD values for the constituent atoms fluctuate around a distinct average, illustrated in Figs. S4 -S6. In direct comparison to $LiFePO_4$ (see Fig. S1-S3) all atoms are less mobile, displaying lower average RMSD values for each atom type. Li^+ ions within $LiMnPO_4$ are the most mobile, with a clear separation in RMSD value in comparison to the rest of the structure.

S3. Li Diffusion Mechanisms

S3.1 $LiFePO_4$ – Low Shooting Regime

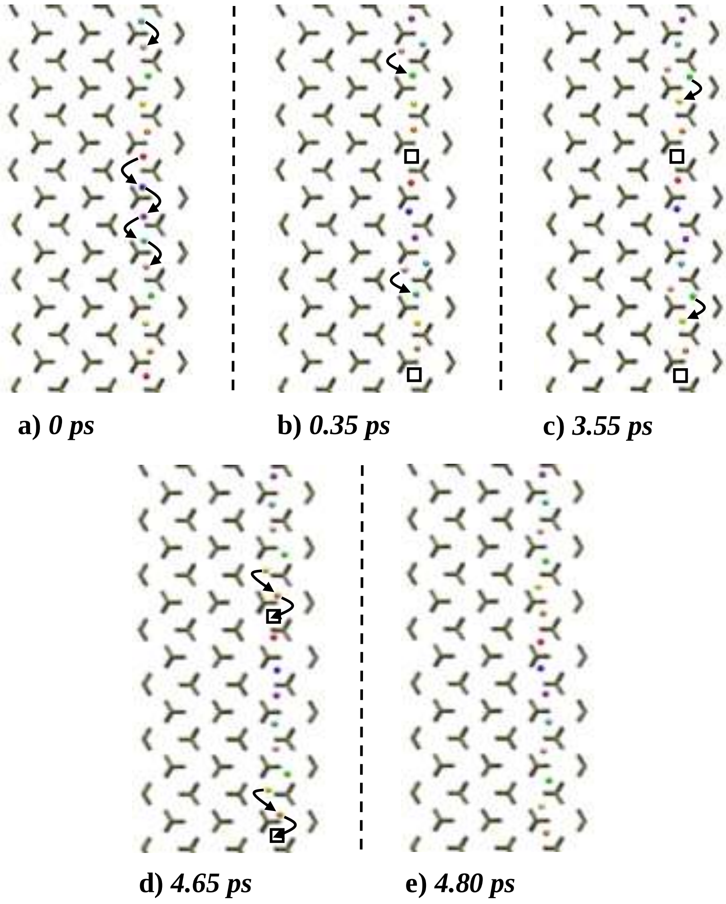

Figure S10: Snapshots of a representative sequence mechanism along [010] in $LiFePO_4$. All Li^+ are individually colored (periodic images are same-colored). The channel is first activated by the formation of a Frenkel defect (a), which occurs via the combination of multiple Li^+ ion jumps. Vacancy (square) and double occupancy begin to migrate down the channel via single Li^+ ion jumps (b, c) until recombination (e). As a result, the column of Li^+ ions have moved down one crystallographic site (e).

S3.2 $LiFePO_4$ – High Shooting Regime

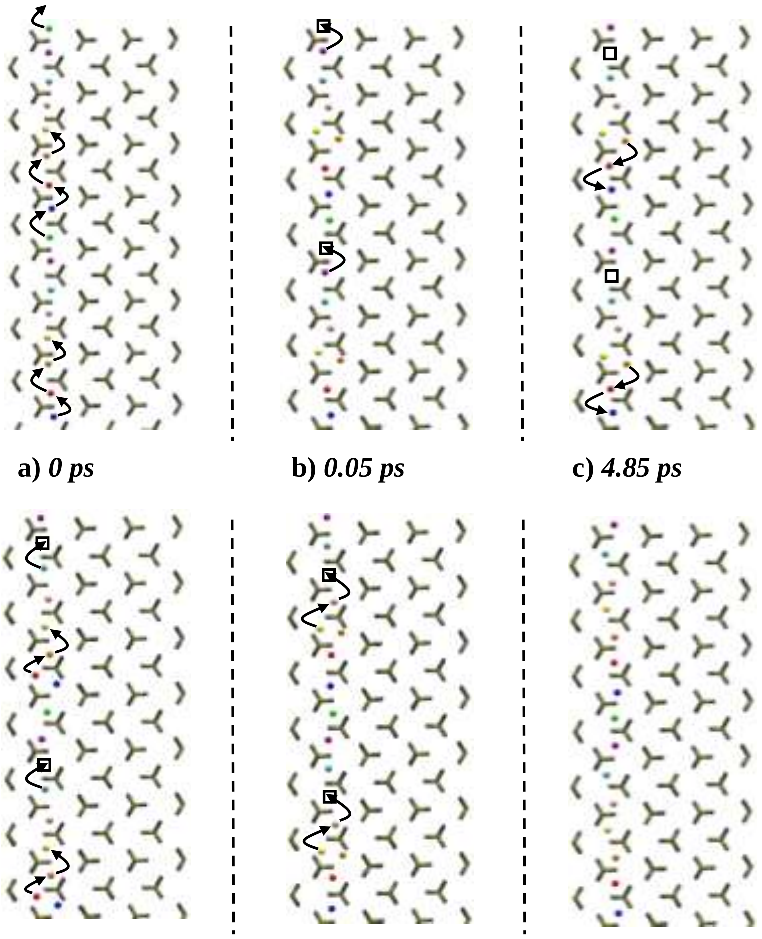

a) ***0 ps*** **b)** ***0.05 ps*** **c)** ***4.85 ps***

d) ***6.00 ps*** **e)** ***23.90 ps*** **f)** ***25.00 ps***

Figure S11: Snapshots of a typical diffusion mechanism within the [010] channel of $LiFePO_4$. Li^+ ions are individually colored under consideration of periodic images (same-color coded).

The channel is first activated by the formation of a Frenkel defect (a), which emerges from the co-operative displacement of multiple single Li^+ ions. The vacancy then migrates *via* a single event (b). Due to the metastable nature of the double-occupancy, this also migrates along the channel through a series of coupled jumps (c, d, e). Finally, the Frenkel pair recombine resulting in the columns of Li^+ ions shifting upwards by one crystallographic site. This mechanism substantially compares with the one obtained in a low shooting regime, see Fig. S9.

S3.3 $LiFePO_4$ – Low Shooting Regime ([001] crossings)

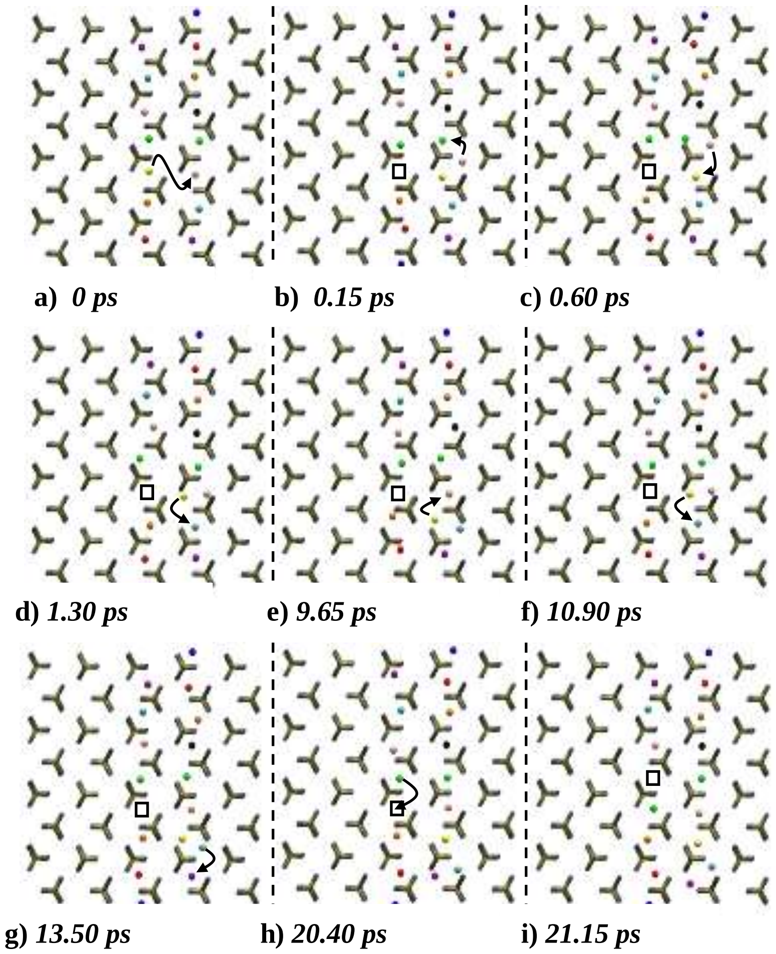

Figure S12: Snapshots of [001] cross-channel migration and subsequent events along [010]. Li^+ ions are individually colored (same color for periodic images). After an initial [001] cross-channel jump (a), a Frenkel defect is formed *across* channels (b). A single Li^+ ion (right) initiates multiple jumps along [010] (b, c, d, f and g). The presence of a vacancy in the left channel also facilitates diffusion (h), migrates away from the double occupied site, and stays separated from it (i).

S4. Mean Squared Displacements

S4.1 $LiFePO_4$ – Low Shooting Regime

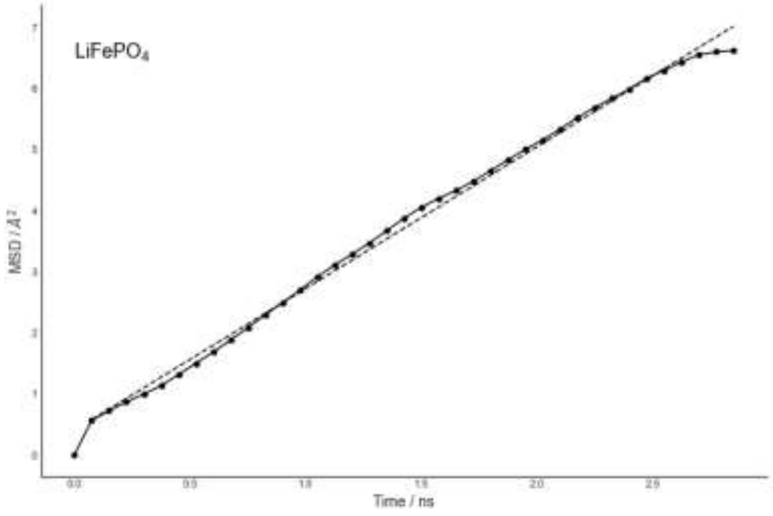

Figure S13: $LiFePO_4$ (low). MSD vs time for all Li^+ ions. MSD is averaged over multiple time origins. ω_f = 0 ns, ω_t = 3.7 ns and ω_s = 75 ps. $D_s^{Li} = 3.87 \pm 0.19 \times 10^{-8}\ cm^2 s^{-1}$.

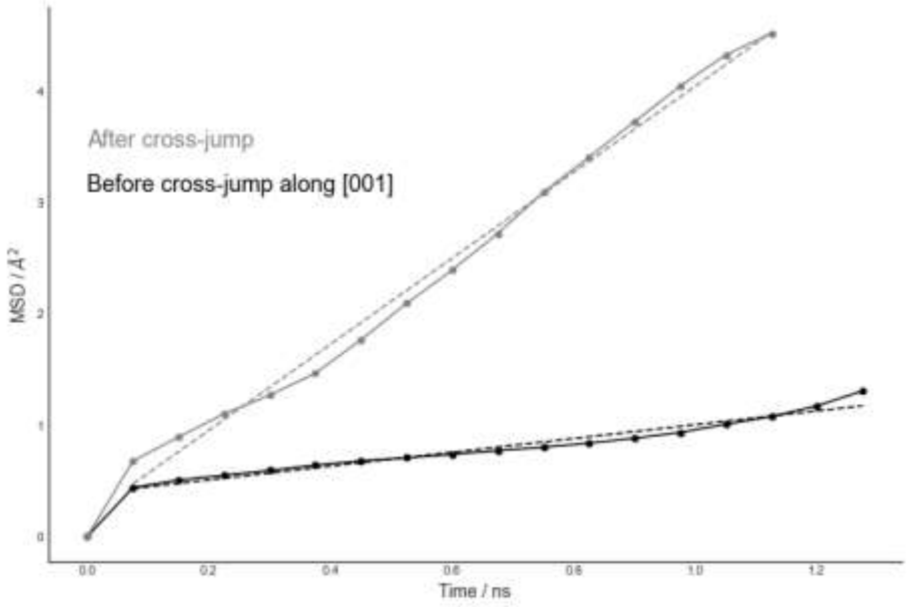

Figure S14: $LiFePO_4$ (low). MSD vs. time for all Li^+ ions before the cross-jump event (black) and after (grey). $D_s = 1.03 \pm 0.08 \times 10^{-8}\ cm^2 s^{-1}$(before) and $D_s = 6.45 \pm 0.20 \times 10^{-8}\ cm^2 s^{-1}$(after).

S4.2 $LiFePO_4$ – High Shooting Regime

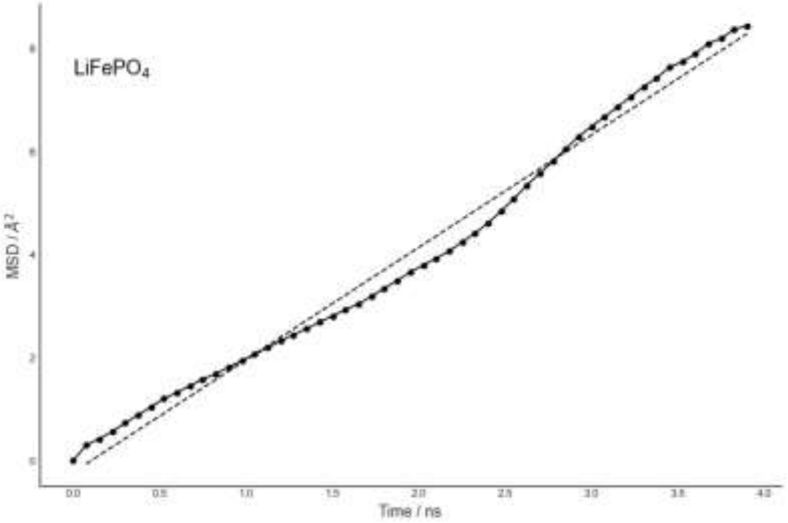

Figure S15: $LiFePO_4$. MSD vs. time, averaged over multiple time origins. ω_f = 0 ns, ω_t = 4.12 and ω_s = 75 ps. D_s = 3.65 $\pm$ 0.45 $\times$ 10^{-8} cm^2s^{-1}.

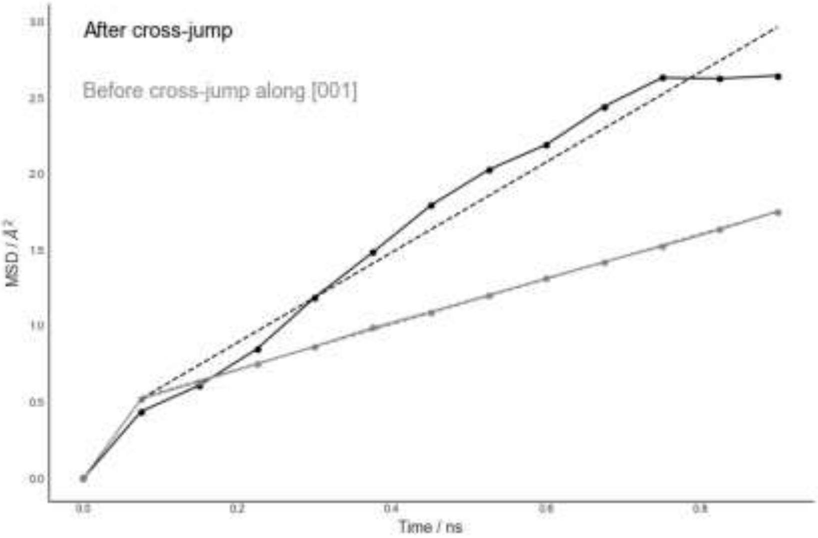

Figure S16: LiFePO4. MSD vs time for all Li^+ ions before the cross-jump along [100] (grey) and after (black). D_s = 2.47 $\pm$ 0.01 $\times$ 10^{-8} cm^2s^{-1}(before) and D_s = 4.94 $\pm$ 0.20 $\times$ 10^{-8} cm^2s^{-1}(after).

S4.3 $LiMPO_4$ – "Optimized" Shooting Regime

MSD and diffusion constants were determined from shooter simulations using a shooting delay of 0.5 ps and Gaussian half-width of 0.0001 Å^2/fs. This choice produced a steadier ion migration profile, allowing for additional precision in the determination of diffusion constants.

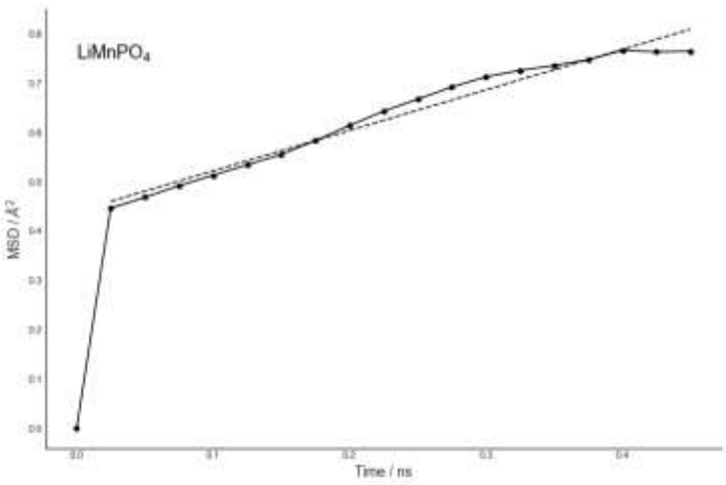

Figure S17: $LiMnPO_4$. MSD vs time for all Li^+ ions. MSD averaged over multiple time origins. ω_f = 0.5 ns, ω_t = 1 ns and ω_s = 25 ps. The self-diffusion coefficient is D_s = 1.28 $\pm$ $0.02 \times 10^{-8} cm^2 s^{-1}$.

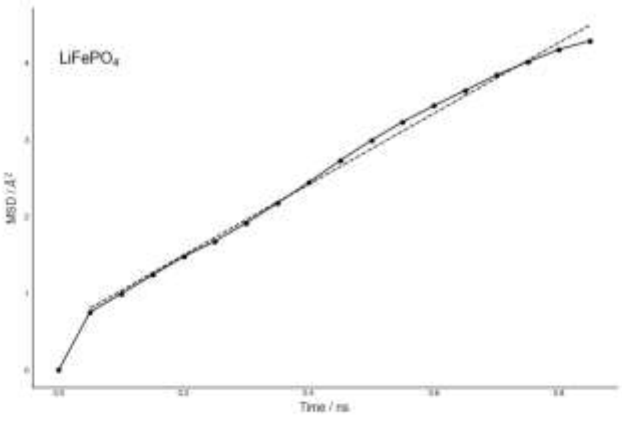

Figure S18: $LiFePO_4$. MSD vs time for all Li^+ ions. MSD is averaged over multiple time origins. ω_f = 0 ns, ω_t = 1 ns and ω_s = 50 ps. The self-diffusion coefficient is D_s = 8.01 $\pm$ $0.08 \times 10^{-8} cm^2 s^{-1}$.

S4.4 $LiFePO_4$ – Antisite Defects (Optimized Shooting Regime)

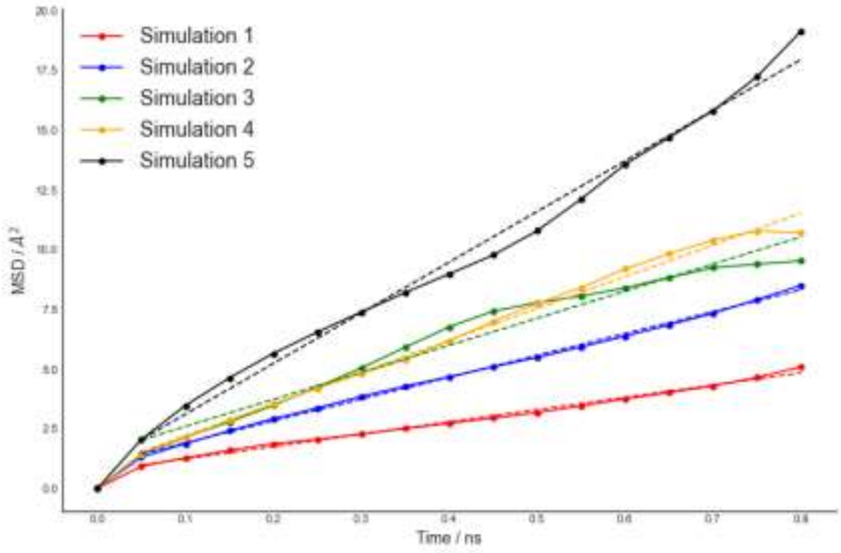

Figure S19: MSD vs time for all Li^+ ions in all five simulations. MSD averaged over multiple time origins. ω_f = 0, ω_t = 1 ns and ω_s = 50 ps. $D_{s,av}$ = 2.01 $\pm$ 0.08 $\times 10^{-7} cm^2 s^{-1}$.

S5 Tight-Binding Molecular Dynamics

Constrained NVT MD simulations were run based on a collective variable (CV), represented by the distance of a Li^+ ion from a “plane” cutting through [010] channels, defined from 3 M atoms (either Fe or Mn) surrounding the channel. A single Li^+ ion was restrained by a harmonic potential between two interstitial sites, “above” and “below” the plane. The resulting sequence of ion displacements is summarized below for $LiFePO_4$ and $LiMnPO_4$.
Force calculations were based on a transferable, semiempirical third-order tight-bind potential, GFN-xTB [S1] as implemented in CP2K. As a variant development of DFTB3 [S2], xTB allows for the description of complex systems containing several interaction types, including covalent, ionic, and dispersive interactions. As a reactive potential, bond-breaking and complicated atomic rearrangements can be reliably and efficiently accounted for. The integration step was 0.2 fs, the temperature of the Nose thermostat was chosen identical to the shooter simulations, 700 K. Electrostatic interactions were accounted for with an Ewald summation, while D3 dispersion correction was used. Slater orbitals were expanded in GTO (Gaussian Type Orbitals) up to sixth order. All other parameters were left at their default values. The simulation box contained 168 atoms (24 Li ions).

S5.1 $LiFePO_4$

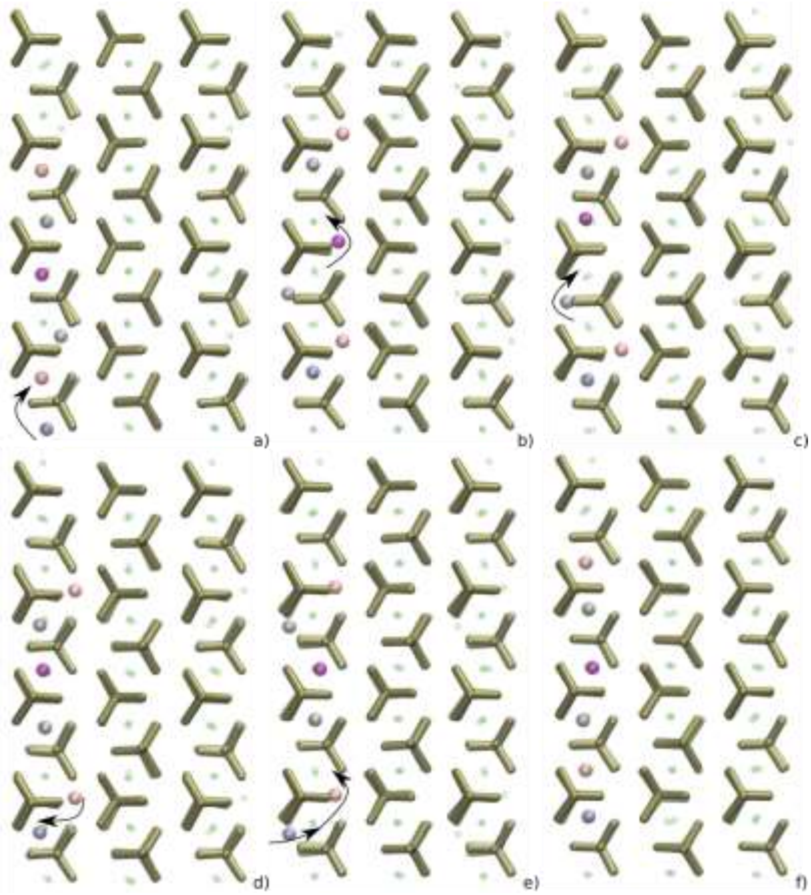

Figure S20: Sequence of [010] in-channel displacements in $LiFePO_4$ in response to the shift of a single Li^+ ion, grey atom in a). The formation of Frenkel defects followed by Li^+ ion

rearrangements (b-e) leads to an overall displacement of Li^+ ions by one site, “upwards”. Li ions are individually colored (same color for periodic images).

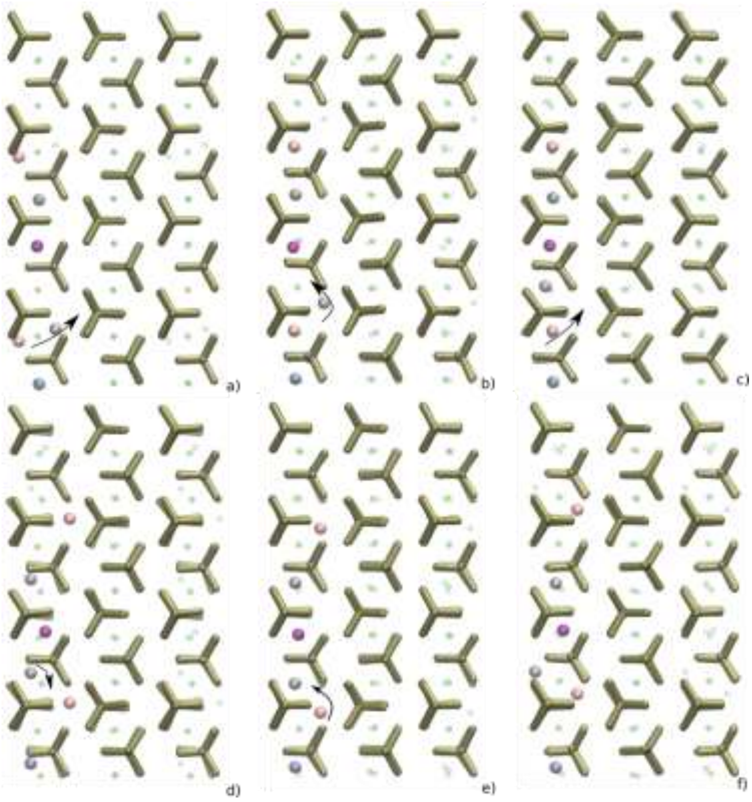

Figure S21: Details of the mobility of a single Frenkel defect along [010] in $LiFePO_4$. From double site occupancy (a), a Li^+ ion is promoted to an adjacent site (c) via intermediate configuration b), followed by double occupancy (d-f). Li ions are individually colored.

S5.2 $LiMnPO_4$

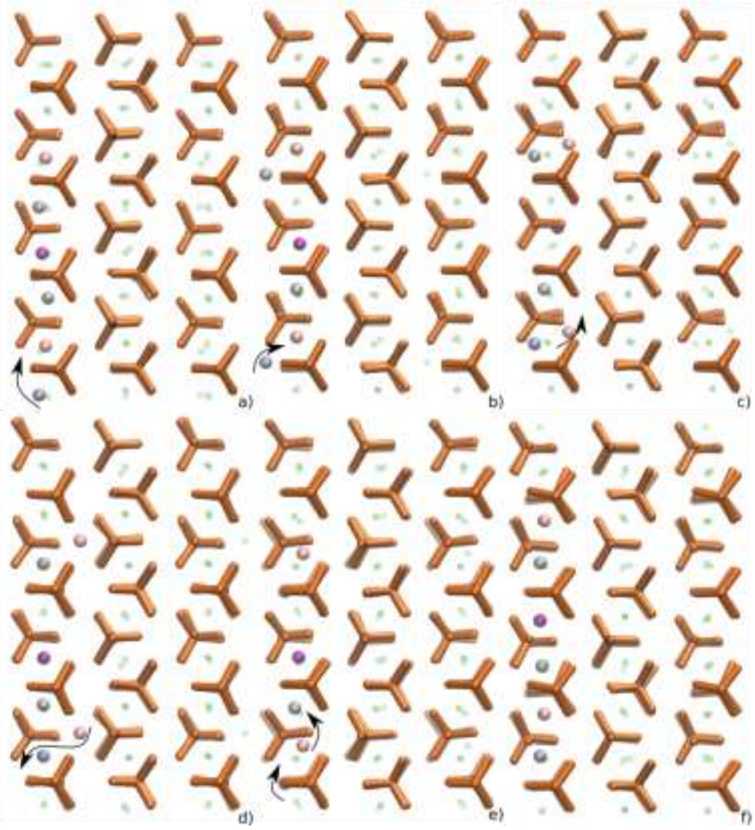

Figure S22: Sequence of [010] in-channel displacements in $LiMnPO_4$, in response to the bias of a single Li^+ ion, lowest grey atom in a). The formation of Frenkel defects (b) followed by Li^+ ion displacement sequences (b-e) leads to an overall displacement of the Li^+ ion by one site, “upwards”, f). Li ions are individually colored (same color for periodic images).

References

[S1] Grimme, S.; Bannwarth, C.; Shushkov, P. A robust and accurate tight-binding quantum chemical method for structures, vibrational frequencies, and noncovalent interactions of large molecular systems parametrized for all spd-block elements (Z= 1–86). *J. Chem. Theory Comput.* **2017**, *13,* 1989– 2009

[S2] Gaus, M.; Cui, Q.; Elstner, M. DFTB3: Extension of the Self-Consistent-Charge Density-Functional Tight-Binding Method (SCC-DFTB). *J. Chem. Theory Comput.* **2011**, *7*, 931– 948.
